# Microbiota-induced peritrophic matrix regulates midgut homeostasis and prevents systemic infection of malaria vector mosquitoes

**DOI:** 10.1371/journal.ppat.1006391

**Published:** 2017-05-17

**Authors:** Faye H. Rodgers, Mathilde Gendrin, Claudia A. S. Wyer, George K. Christophides

**Affiliations:** Vector Immunogenomics and Infection Laboratory, Department of Life Sciences, Imperial College London, London, United Kingdom; Johns Hopkins University, Bloomberg School of Public Health, UNITED STATES

## Abstract

Manipulation of the mosquito gut microbiota can lay the foundations for novel methods for disease transmission control. Mosquito blood feeding triggers a significant, transient increase of the gut microbiota, but little is known about the mechanisms by which the mosquito controls this bacterial growth whilst limiting inflammation of the gut epithelium. Here, we investigate the gut epithelial response to the changing microbiota load upon blood feeding in the malaria vector *Anopheles coluzzii*. We show that the synthesis and integrity of the peritrophic matrix, which physically separates the gut epithelium from its luminal contents, is microbiota dependent. We reveal that the peritrophic matrix limits the growth and persistence of *Enterobacteriaceae* within the gut, whilst preventing seeding of a systemic infection. Our results demonstrate that the peritrophic matrix is a key regulator of mosquito gut homeostasis and establish functional analogies between this and the mucus layers of the mammalian gastrointestinal tract.

## Author summary

When a female mosquito takes a blood meal from a human, the bacteria residing within its gut grow significantly. Following a blood meal, female mosquitoes produce a barrier within their gut, known as the peritrophic matrix, which physically separates the blood meal from the cells of the epithelium. Here, we show that the presence of bacteria in the gut is required for the synthesis of the peritrophic matrix. By experimentally disrupting this barrier, we find that this structure plays a role in limiting the extent to which bacteria of one particular family are able to grow and persist in the mosquito gut. We also find that the peritrophic matrix ensures that bacteria remain within the gut, preventing them from invading the mosquito body cavity. These results will be useful in designing disease control strategies that depend on the ability of bacteria to colonize and persist in relevant tissues in the mosquito host.

## Introduction

Mosquitoes of the *Anopheles* genus are responsible for the transmission of *Plasmodium* parasites, the causative agents of malaria. The study of the *Anopheles* gut microbiota has recently emerged as an important field in an effort to characterize mosquito-parasite interactions in greater depth and to develop new methods to stop disease transmission. The microbiota have been shown to trigger a constitutive immune response in the mosquito gut epithelium that enhances resistance to parasite infection [1,2]. Furthermore, specific gut bacteria have been found to directly impact parasites, compromising their infectivity [3,4]. Finally, a promising transmission-blocking intervention is paratransgenesis, which aims to use vector-associated bacteria as a delivery tool for antimalarial effectors [5]. The success of such an approach would require the persistence of genetically-modified bacteria at sufficient abundance within the gut ecosystem, and potentially their successful dissemination throughout the mosquito body. As such, deeper understanding of mosquito-microbiota interactions may highlight mechanisms by which bacteria can be utilized to block malaria transmission.

The balance between immune resistance and tolerance is key to bacterial persistence within the gut environment. Resistance refers to bacterial killing or the prevention of bacterial growth, whilst tolerance encompasses the prevention or repair of host tissue damage caused by pathogens or immune responses [6]. In *Drosophila*, commensals are controlled largely by the production of reactive oxygen species (ROS) by the dual oxidase (DUOX) enzyme [7,8], whilst the other main resistance mechanism in the *Drosophila* gut, the Imd pathway, is under strong negative regulation to prevent its stimulation by commensals [9–11]. In mosquitoes, blood feeding triggers substantial microbiota proliferation [12,13] and induces high levels of oxidative stress, potentially precluding further production of ROS for immune control [14]. In *A*. *gambiae*, commensals are known to induce the Imd pathway, and suppression of the Imd pathway receptor PGRPLC and its transcription factor REL2 causes microbiota overgrowth [1,2]. The transcription factor Caudal, which is specifically expressed in the gut, down-regulates REL2-dependent expression of antimicrobial peptides (AMPs), facilitating microbiota tolerance [15].

Some tolerance mechanisms are based on the strengthening of physical barriers between the microbiota and the host. Notably, an *A*. *gambiae* heme peroxidase is induced by blood feeding and, together with DUOX, forms a network of dityrosine bonds that is thought to protect the gut epithelium from immune elicitors, thus mediating bacterial persistence [12]. The peritrophic matrix has also been identified as playing a role in host-bacteria interactions in a number of insects. It is an acellular structure composed of chitin, proteins and glycoproteins located between the gut lumen and the epithelium. The mosquito type I peritrophic matrix is produced by adult female midgut cells during blood feeding and physically surrounds the blood bolus, whilst the type II peritrophic matrix is permanently produced by the cardia in the anterior larval gut. The type II peritrophic matrix of the hematophagous tsetse fly provides infectious *Serratia* bacteria with a protective niche in which they can proliferate without inducing a gut immune response, increasing susceptibility to infection [16]. In the tick *Ixodes scapularis*, the gut microbiota induces the formation of a peritrophic matrix whose presence facilitates colonization of the spirochete bacterium *Borrelia burgdorferi*, possibly by protecting the pathogen from blood meal pro-oxidants and cellular immunity [17]. In *Drosophila*, oral bacterial infection induces the expression of genes encoding proteins with chitin binding domains (CBDs) [18], and a protein of the type II peritrophic matrix is shown to reduce both local and systemic Imd pathway stimulation and to protect epithelial cells against pore-forming toxins [19,20].

The mosquito peritrophic matrix is often considered as a barrier to parasite infection, though one that parasites have evolved to overcome. Secretion of chitinase by *Plasmodium* effectively facilitates traversal of the peritrophic matrix [21–25]. A constitutive peritrophic matrix protein, fibrinogen-related protein 1 (FREP1), has recently been proposed to be exploited by invading parasites, serving as an anchor that facilitates *P*. *falciparum* invasion [26]. More generally, the *Aedes aegypti* peritrophic matrix is thought to play a role in blood meal detoxification, sequestering large quantities of heme released during blood bolus digestion [27]. The role of the mosquito peritrophic matrix in bacterial pathogenesis and microbiota homeostasis in the gut has not yet been explored.

Here, we use RNA sequencing to explore the microbiota-dependent gene expression in the midgut of the *A*. *coluzzii* mosquito (until recently known as *A*. *gambiae* M form). We find that the gut microbiota induce the expression of several components of the peritrophic matrix, and that the microbiota are necessary for the synthesis of a structurally complete peritrophic matrix. We also show that the peritrophic matrix plays a role in resistance to the *Enterobacteriaceae* bacteria present in the gut microbiota, both reducing the extent to which this family of bacteria grows and persists within the gut, and precluding this family of bacteria from seeding a systemic infection.

## Results

### RNA sequencing analysis of microbiota-dependent expression in the gut

**Fig 1:**
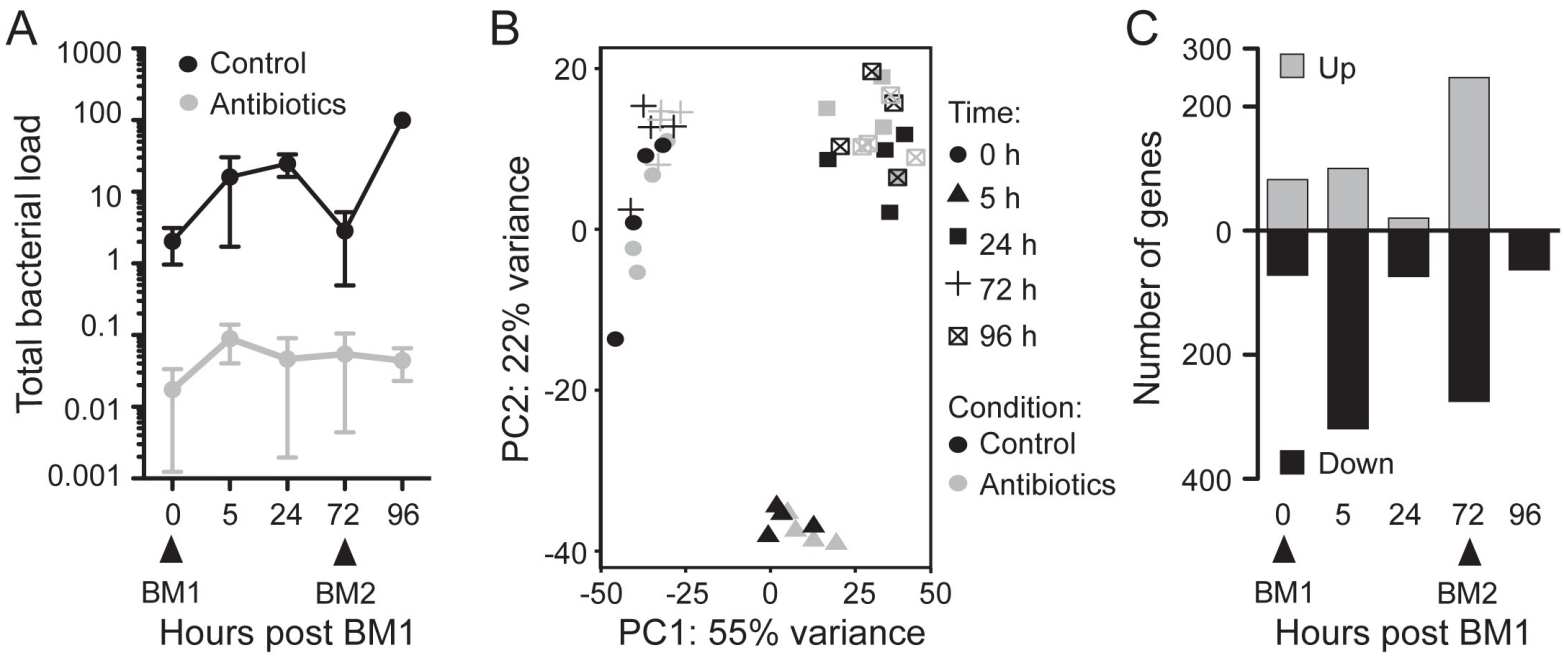
Effect of oral antibiotic treatment on the gene expression of the mosquito midgut. **(A)** Bacterial load in the guts of 4 control and 4 antibiotic treated mosquito cohorts throughout a two blood meal (BM1 and BM2) time course assessed by qRT-PCR using universal 16S primers. Data were normalized within each biological replicate to the bacterial load in the control 96h sample. The mean plus/minus the standard error is shown. **(B)** Principal components analysis (PCA) plot of the 40 sequenced midgut samples after variance stabilizing transformation of count data. **(C)** Number of genes that were significantly upregulated (white bars) or downregulated (black bars) in each of the time points following antibiotic treatment. Genes showing an adjusted p-value <0.1 (Wald test with a Benjamini-Hochberg correction) were considered to be significantly regulated.

To explore the transcriptional response to the dynamic changes in microbiota load over the blood feeding cycle, we sequenced RNA extracted from *A*. *coluzzii* midguts at five time points: 2–3 day old mosquitoes that had accessed only fructose since emergence (sugar-fed, ‘SF’), 5h, 24h and 72h after a human blood meal and 24h (96h) after a second human blood meal that was given at the 72h time point. This time course was performed with conventionally-reared mosquitoes that harbored their native microbiota, and a cohort of mosquitoes that were fed an antibiotic cocktail (50μg/ml gentamicin, 60μg/ml streptomycin, 60U/ml penicillin) in both sugar and blood meals. This antibiotic treatment was effective in substantially depleting mosquito guts of bacteria as detected by qRT-PCR against 16S rRNA (Fig 1A). Each sample consisted of a pool of 20 midguts, and four independent replicates were performed using four independent batches of mosquitoes, as there is evidence that the microbiota of laboratory-reared mosquitoes varies between generations [1]. The resulting cDNA libraries were sequenced across four lanes of an Illumina flowcell on an Illumina Hiseq 1500, resulting in a total of 893,247,801 pairs of 100bp reads across the forty samples. After quality control, an average of 85.6% of input sequences per sample aligned uniquely to the *A*. *gambiae* PEST genome (AgamP4). A total of 6753 genes (49.9% of all annotated genes) had non-zero counts in all forty samples. Principal component analysis (PCA) indicated that the samples clustered according to their blood feeding status, with no obvious outliers (Fig 1B).

Soft clustering analysis indicated that the oral antibiotic treatment had an overall relatively minor effect on the general transcriptional changes occurring over the blood feeding cycle (S1 Fig). Nevertheless, we identified 889 genes that were significantly differentially regulated at one or more time points by antibiotic treatment (S1 File). Gene Ontology (GO) and Kyoto Encyclopedia of Genes and Genomes (KEGG) enrichment analysis implicated these genes in diverse processes, including carbohydrate, protein and lipid metabolism, folate biosynthesis, oxidation-reduction processes and immunity (S1 Table). Interestingly, the 0h and 72h samples exhibited the greatest number of differentially regulated genes (Fig 1C), despite having the lowest bacterial load in the control samples. We hypothesised that this could be indicative of the microbiota playing a more significant role in midgut physiology at these time points, or of the existence of highly effective tolerance mechanisms in the gut following blood feeding.

### Peritrophic matrix synthesis is microbiota-dependent

**Fig 2:**
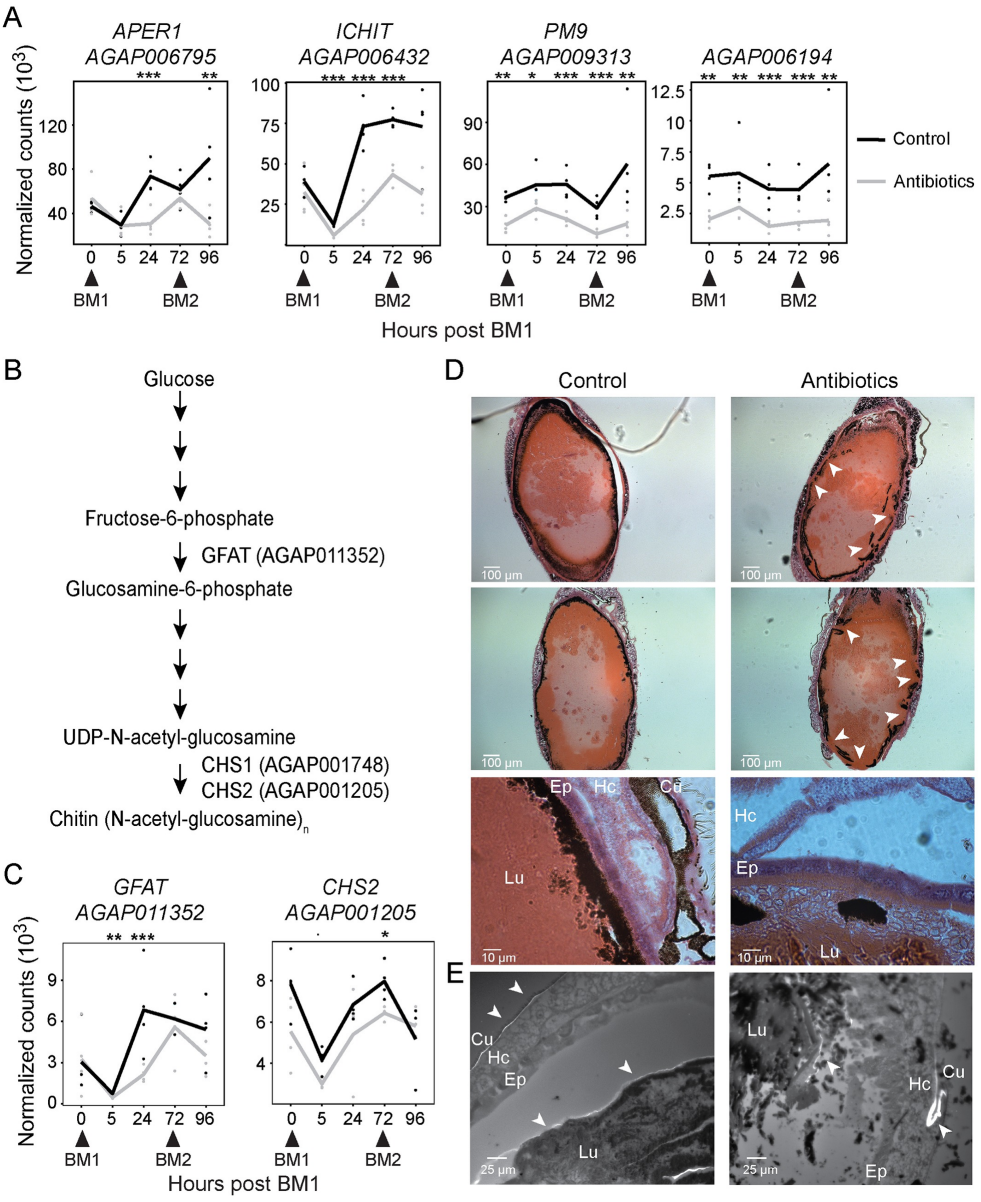
Antibiotic treatment compromises the production and integrity of the peritrophic matrix. **(A)** Transcriptional profiles of four genes encoding putative components of the peritrophic matrix in the midguts of control (black line) and antibiotic-treated (grey line) mosquitoes over a course of two consecutive blood meals (BM1 and BM2). Dots indicate normalized counts of each of four biological replicates, with the lines connecting the means. Statistical significance of a pairwise comparison of counts at each time point using the Wald test with Benjamini-Hochberg correction is indicated: ^**‘.’**^ p<0.1; ‘*’ p<0.05; ‘**’ p<0.01; ‘***’ p<0.001 **(B)** Schematic of the chitin synthesis pathway in insects. Gene IDs indicate genes in the *A*. *gambiae* PEST genome annotated with the indicated enzyme activity. GFAT; glucosamine-fructose-6-phosphate aminotransferase, CHS1; chitin synthase 1, CHS2; chitin synthase 2. **(C)** Transcriptional profiles of the GFAT and CHS2 encoding genes during the course of two consecutive blood meals (BM1 and BM2) following antibiotic-treated (grey line) compared to non-treated (black line) controls. Dots and stars indicate counts and p-values as explained in (A). **(D)** H&E stained thin sections of engorged midguts 24h post blood feeding with or without antibiotic supplementation. Arrowheads indicate regions of apparent disruption of the PM integrity. **(E)** Calcofluor white stained thin sections of engorged mosquitoes 24h post blood feeding with or without antibiotic supplementation. Arrowheads indicate staining of the abdominal cuticle and the peritrophic matrix. For (D) and (E): “Lu” lumen; “Ep” epithelium; “Hc” hemocoel; “Cu” cuticle.

As observed previously [1], we noted that several microbiota-regulated genes encoded proteins containing CBDs, a signature of the structural components of the peritrophic matrix. Although the precise structure of the peritrophic matrix remains under-explored, a proteomic analysis has previously identified its most abundant protein components [28]. Of genes encoding 24 of the top candidate proteins identified in that study, 12 were significantly differentially regulated in our dataset, with 11 of these being down regulated following antibiotic treatment (S2 Table). The microbiota-induced genes included *AgAPER1* (AGAP006795; Fig 2A), which encodes a chitin-binding *A*. *gambiae* peritrophic matrix component [29] and was the most abundant CBD-containing protein identified by mass spectrometry [28]. We also noted the microbiota-dependent expression of *ICHIT* (AGAP006432) that is known to be transcriptionally induced by both *P*. *berghei* and bacterial infections and encodes two CBDs and a proline-rich domain that may be involved in protein-protein aggregation [30] (Fig 2A). Two genes (AGAP009313 and AGAP006194) encoding proteins identified in the peritrophic matrix proteomic study [28] were significantly microbiota regulated at all five time points (Fig 2A); neither of these genes encode CBD-containing proteins.

In addition to the protein components of the peritrophic matrix, the main structural constituent is chitin, a polymer of N-acetylglucosamine. Insects are able to synthesize chitin from glucose in a multistep reaction (Fig 2B); fructose-6-phosphate, derived from glucose, is converted to glucosamine-6-phosphate in a rate limiting step catalyzed by glucosamine-fructose-6-phosphate aminotransferase (GFAT) [31]. Glucosamine-6-phosphate is then metabolized to UDP N-acetylglucosamine, which is subsequently polymerized to chitin fibers by chitin synthase. The *A*. *gambiae* genome encodes two chitin synthase enzymes, CHS1 (AGAP001748) and CHS2 (AGAP001205), of which CHS2 is expressed in the midgut and responsible for the synthesis of peritrophic matrix-associated chitin [32]. Here, we identified GFAT and CHS2, the enzymes catalyzing two rate-limiting steps of the chitin synthesis pathway, as being microbiota-regulated at the transcript level at one or more of the time points examined (Fig 2C). Following antibiotic treatment the expression of these enzymes is either significantly reduced or temporally delayed.

We speculated that this transcriptional response in the production of both chitin and peritrophic matrix proteins could be bacterially induced either directly, through detection of bacterial elicitors, or indirectly as a result of bacteria causing thinning of the peritrophic matrix (e.g. through chitinases) that results in compensatory transcription to produce a peritrophic matrix of normal thickness. We used thin abdominal sections 24h post blood meal stained with hematoxylin and eosin (H&E) to observe the effect of oral antibiotic treatment on the structure of the gut tissue (Fig 2D). In the control samples, a thick layer of dark pigment was observed surrounding the blood bolus and effectively separating the contents of the lumen from the epithelial cells. We hypothesized that this dark layer is likely an accumulation of heme pigment at the surface of the peritrophic matrix. Indeed, heme released during hemoglobin digestion is shown to bind CBD-containing peritrophic matrix proteins in *Aedes aegypti* mosquitoes [27,33,34]. In the antibiotic-treated mosquitoes, several regions exhibited disruption of this layer, suggestive of a similar disruption of the peritophic matrix, resulting in red blood cells (RBCs) coming into direct contact with the epithelial cells (Fig 2D). Quantification of such instances confirmed that RBC contact with the epithelium is significantly increased in antibiotic-fed (92%, n = 12) compared to control guts (6%, n = 18; p<0.0001, Chi-square test). To confirm the disruption of the peritrophic matrix, we stained abdominal sections with the chitin specific stain calcofluor white (Fig 2E). In both control and antibiotic treated samples, chitin specific staining of the cuticle was observed. In control mosquitoes, we additionally observed a prominent layer of chitin staining surrounding the blood bolus, which corresponds to the peritrophic matrix. In the antibiotic treated group this staining was either absent or fragmented. These observations suggest that, indeed, the presence of the microbiota is required for the synthesis of a structurally complete peritrophic matrix.

### The peritrophic matrix regulates resistance to the microbiota

The RNAseq data indicated that the microbiota play a significant role in regulating antimicrobial peptide (AMP) expression in the gut, with seven characterized immune effector-encoding genes being upregulated at one or more time points by the presence of the microbiota (S3 Table). These AMPs include three cecropins (*CEC1*, *CEC2* and *CEC3*), one defensin (*DEF1*), gambicin (*GAM1*) and two C-type lysozymes (*LYSC1* and *LYSC7*). We therefore sought to explore whether the peritrophic matrix plays a role in mediating the mosquito immune response to the microbiota. We supplemented the blood meal with 100μM polyoxin D, a chitin synthase inhibitor that has previously been demonstrated to abolish synthesis of the *A*. *gambiae* type I peritrophic matrix [24]. Staining of abdominal mosquito sections with calcofluor white at 24h post blood meal confirmed the absence or fragmentation of the peritrophic matrix upon treatment with polyoxin D (S2A Fig). In the midguts of polyoxin D-fed mosquitoes, we also observed increased RBC contact with the epithelium (47%, n = 17) compared to control guts (19%, n = 32; p<0.05, Chi-square test; S2B Fig).

**Fig 3:**
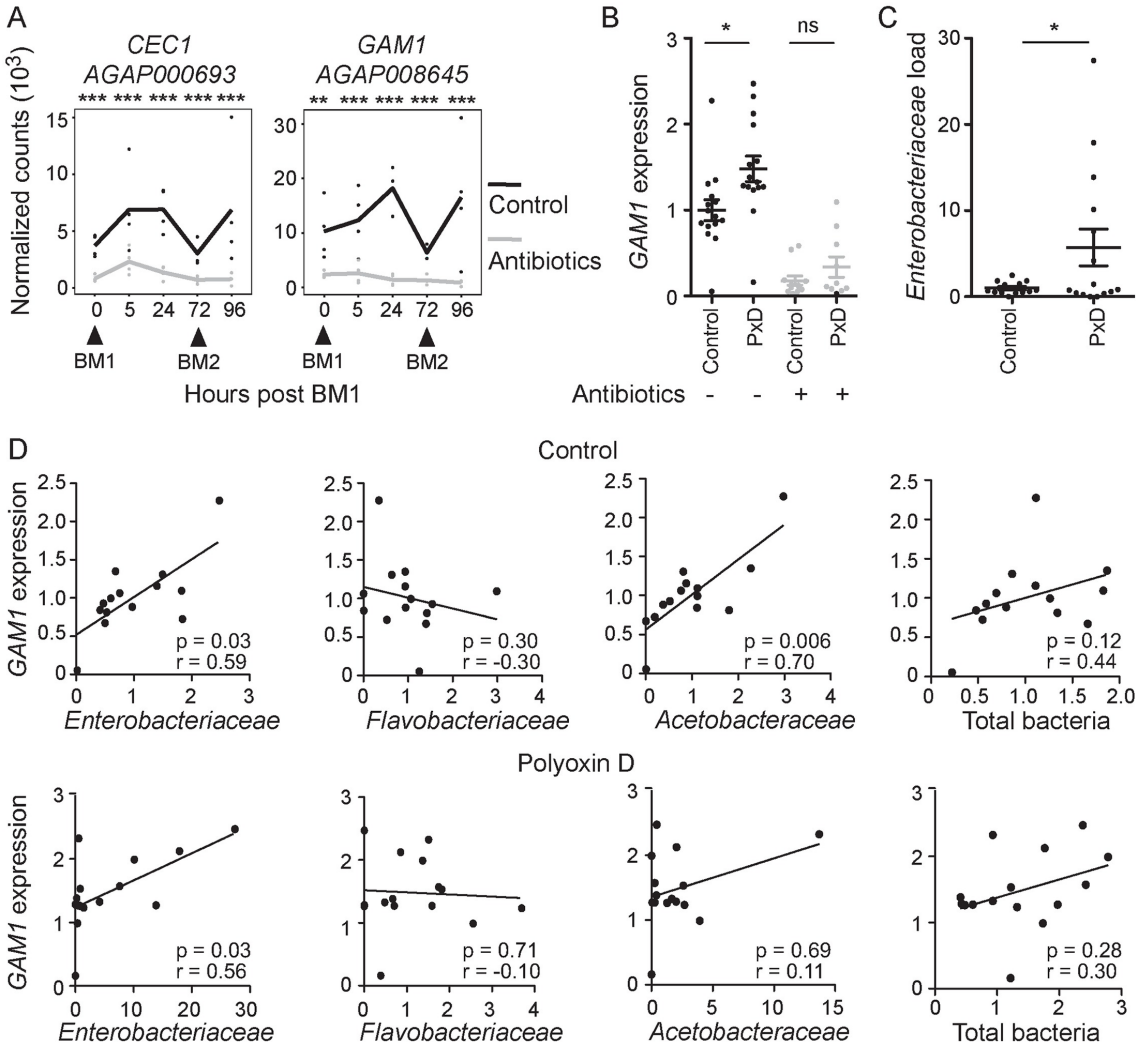
The peritrophic matrix regulates immune resistance to the microbiota. **(A)** RNA-seq transcriptional profiles of *CEC1* and *GAM1* in the midgut of control (black lines) and antibiotic fed (grey lines) mosquitoes over a two blood meal (BM1 and BM2) time course. Dots indicate normalized counts in each of four biological replicates, with the line connecting the means. Statistical significance of a pairwise comparison of counts at each time point was assessed by a Wald test with a Benjamini-Hochberg correction. ‘*’ p<0.05; ‘**’ p<0.01; ‘***’ p<0.001 (**B)** *GAM1* expression, relative to AgS7, in the midgut 24h after feeding with blood supplemented with 100μM polyoxin D (PxD) or an equal volume of water (control), plus or minus antibiotic treatment, as determined by qRT-PCR. Mean plus/minus standard error is indicated. Statistical significance was assessed by an ANOVA on a linear mixed effect regression model. Each dot represents a pool of 8–10 guts, derived from 4–5 independent experiments. Ratios are normalized within biological replicates to the mean of the control pools (no polyoxin D, no antibiotics). **(C)** *Enterobacteriaceae* load, relative to AgS7, as determined by qRT-PCR using *Enterobacteriaceae* specific 16S primers. Normalization and statistical analysis were performed as described for (B). **(D)** Scatter plots of the load of specific bacteria families commonly found in the mosquito gut against *GAM1* expression in the midguts of control (top row) or polyoxin D-treated (bottom row) mosquitoes. Spearman’s rank correlation coefficient (r) and associated p-values (p) are indicated.

We next investigated whether the peritrophic matrix plays a role in modulating the midgut epithelium response to the microbiota. We selected two AMP reporter genes, *CEC1* and *GAM1*, which showed strong microbiota-dependent expression at all time points examined (Fig 3A and S3 Table). The midgut expression of the two AMP genes was monitored at 24h after blood meal supplementation with 100μM polyoxin D or an equal volume of water as a control. We observed a significant increase in the expression of *GAM1* in the midguts of polyoxin D-treated mosquitoes (Fig 3B), whilst the increase in *CEC1* expression between polyoxin D treated and untreated mosquitoes was not statistically significant (S3A Fig). Treatment of mosquitoes with antibiotics revealed that the increase of *GAM1* expression was microbiota-dependent (Fig 3B). In order to confirm that the effect of polyoxin D on the immune response in the gut was due to disruption of the peritrophic matrix as opposed to any direct effect of polyoxin D on gut bacteria or the cells of the epithelium, we sought an independent method of peritrophic matrix disruption. To this end, we silenced by RNAi the *APER1* gene that encodes an abundant peritrophic matrix component. The results showed an elevated immune response in the midguts of *APER1* knock down mosquitoes, corroborating the polyoxin D feeding experiments (S3B Fig); in this case, *CEC1* expression was significantly increased, whilst *GAM1* expression also showed non-significant up-regulation. This effect was again microbiota dependent (S3B Fig).

These data raised the question whether disruption of the peritrophic matrix affects tolerance or resistance mechanisms. In the former case, the elevated AMP expression in peritrophic matrix-disrupted midguts would reflect increased access of bacteria and immune elicitors to the innate immune receptors found on the epithelial cells, which could consequently result in decreased bacterial growth. In the latter case, disruption of the peritrophic matrix could relieve bacterial growth from biochemical and/or physical constraints, which may result in a higher bacterial load, consequently increasing AMP induction. We quantified the total bacterial load in the polyoxin D-treated compared to control midguts as well as the specific load of three bacterial families commonly found in *Anopheles* midguts: *Enterobacteriaceae*, *Flavobacteriaceae* and *Acetobacteraceae* [35]. We did not detect a significant difference in total bacterial load nor in the load of bacteria of the *Flavobacteriaceae* and *Acetobacteraceae* families between the two groups (S3C Fig). However, a significant increase was detected in the load of the *Enterobacteriaceae*, which was highly variable between midgut pools (Fig 3C). Corroborating this, we also observed substantial *Enterobacteriaceae* overgrowth in a subset of the *APER1* knock down mosquito cohorts compared with the *LACZ* controls (S3D Fig). These data point to a role of the peritrophic matrix in resistance to the *Enterobacteriaceae*.

To further characterize the role of the microbiota in AMP regulation, we examined the correlation between the bacterial loads and *GAM1* expression in each of the mosquito pools used in the polyoxin D experiments described above. In control mosquitoes, *GAM1* expression positively correlated with both the *Enterobacteriaceae* and *Acetobacteraceae* loads, but not with the total bacterial load or the *Flavobacteriaceae* load (Fig 3D). These data suggest that the *Enterobacteriaceae* and *Acetobacteraceae* families are primarily responsible for the induction of *GAM1*. In the polyoxin D-fed mosquitoes, the positive correlation between *Enterobacteriaceae* load and *GAM1* expression was maintained, but not that between *Acetobacteraceae* load and *GAM1* expression (Fig 3D). These data are consistent with a model whereby formation of the peritrophic matrix serves as a resistance mechanism, limiting the growth of the *Enterobacteriaceae* after blood feeding and the extent to which this family of bacteria induces a local immune response.

### The peritrophic matrix contributes to restoration of homeostasis after a blood meal

**Fig 4:**
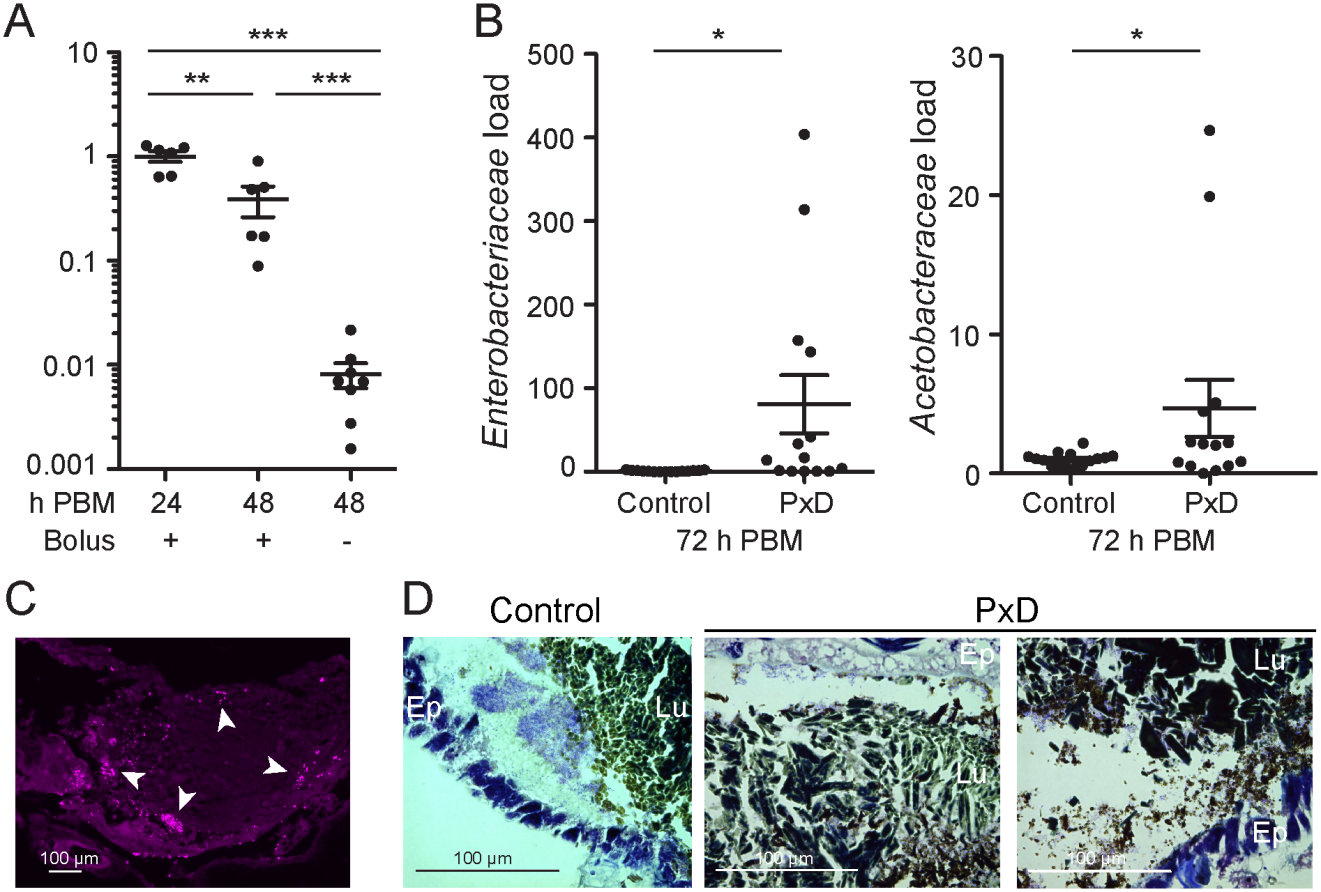
The peritrophic matrix promotes restoration of gut homeostasis after blood feeding. **(A)** Midgut bacterial load at 24h and 48h after a blood meal, as determined by qRT-PCR. At 48h, midguts were pooled according to whether (+) or not (-) the blood bolus was present. Each dot represents a pool of 5 guts, derived from two independent experiments. Ratios are normalized within biological replicates to the mean of the 24h pools. Mean plus/minus standard error is indicated. Statistical significance was assessed by an ANOVA on a linear mixed effect regression model. ‘*’ p<0.05; ‘**’ p<0.01; ‘***’ p<0.001. **(B)** *Enterobacteriaceae* and *Acetobacteraceae* load in midguts 72h after feeding on blood supplemented with 100μM polyoxin D (PxD) or an equal volume of water (control), as determined by qRT-PCR. Each dot represents a pool of 8–10 guts, derived from 4 independent experiments. Ratios are normalized within biological replicates to the mean of the control pools. Mean plus/minus standard error is indicated. Statistical significance was assessed and is presented as described above. **(C)** Thin abdominal section 24h post blood meal stained with anti-LPS antibody. White arrowheads indicate LPS staining. **(D)** Gram-stained thin abdominal sections 24h post blood meal, with or without polyoxin D supplementation. Bacteria are stained light purple. “Lu” lumen; “Ep” epithelium.

By 72h after the blood meal, we observed that gut microbiota load had been restored to pre-blood feeding levels (Fig 1A) [36]. We sought to understand the mechanisms underlying this re-establishment of homeostasis following blood feeding, hypothesizing that the excretion of the blood bolus may additionally facilitate the physical removal of bacteria from the gut. To investigate this, we monitored individual mosquitoes 48h after blood feeding, dividing them into two groups according to whether or not they had excreted their blood bolus, and analyzed the gut bacterial load in each group (Fig 4A). We found that, indeed, mosquitoes that had excreted their blood bolus had 98% lower bacterial load than those that still retained their blood bolus at this time point. These results strongly suggested that bacteria are excreted with the blood bolus, thus contributing to the restoration of gut homeostasis.

Upon completion of blood digestion, the peritrophic matrix is believed to be excreted with the blood bolus [37]. To investigate whether the peritrophic matrix plays a role in mediating bacterial excretion, we monitored the effect of peritrophic matrix disruption on the bacterial load at 72h post blood feeding, a time point at which all individuals have excreted their blood bolus. The polyoxin D-fed cohort of mosquitoes harbored significantly higher loads of *Enterobacteriaceae* and *Acetobacteraceae* than the control cohort (Fig 4B), as well as non-significantly higher loads of *Flavobacteriaceae* and total 16S rRNA (S4 Fig), suggesting that the peritrophic matrix prevents bacteria from occupying niches within the gut that cannot be cleared upon excretion of the blood bolus. We performed immunohistochemistry against lipopolysaccharide (LPS), a major component of the outer membrane of Gram-negative bacteria, to investigate bacterial localization in the mosquito gut. We observed the majority of staining in the periphery of the gut, suggesting possible co-localization of the gut bacteria with the peritrophic matrix (Fig 4C).

We next used Gram staining to investigate this localization further in both control and polyoxin D treated guts. In the control guts, we observed bacteria localizing between the blood bolus and the epithelial cell layer (Fig 4D). In the polyoxin D treated guts, bacterial localization was more diffuse, with bacteria being observed at the periphery of the blood bolus, and indeed proximally to the cells of the epithelium, as well as within the gut lumen, amongst the blood bolus. Together, these data are suggestive of a model whereby the presence of an intact peritrophic matrix facilitates the efficient clearance of bacteria from the gut after blood bolus digestion, while co-localization of the gut bacteria and the peritrophic matrix may be a pre-requisite of this.

### The peritrophic matrix prevents dissemination of the midgut microbiota into the body cavity

**Fig 5:**
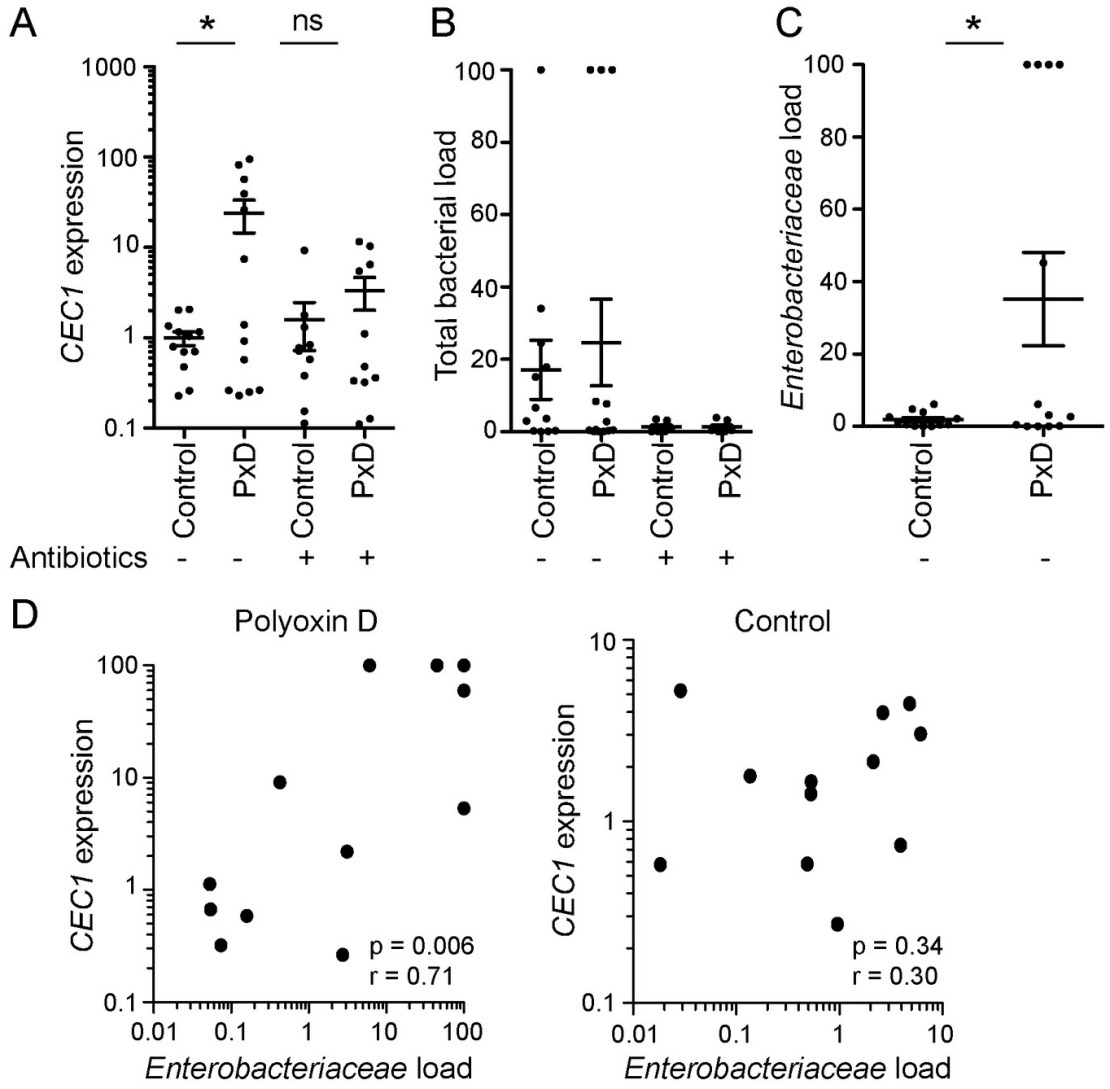
The peritrophic matrix prevents microbiota dissemination and systemic immune induction. **(A-C)** *CEC1* expression (A), *16S rRNA* quantification (B) and *Enterobacteriaceae 16S rRNA* quantification (C) in the carcass 72h after feeding with a blood meal supplemented with 100μM polyoxin D or water as a control, plus or minus antibiotic treatment, as determined by qRT-PCR. Each dot represents a pool of 8–10 carcasses, derived from 4 independent experiments. Data show mean and standard error. In A, ratios are normalized within biological replicates to the mean of the control pools (no polyoxin D, no antibiotics). In B-C, ratios are normalized within each biological replicate to the highest value across all conditions (‘100%’). In A-C, statistical significance was assessed by an ANOVA on a linear mixed effect regression model. **(D)** Scatter plots of relative *Enterobacteriaceae* load against *CEC1* expression in the carcass at 72h post blood feeding. Each dot represents a pool of 8–10 carcasses, derived from 4 independent experiments; data are normalized as in B and C. Spearman’s rank correlation coefficient and associated p-values are indicated. ‘*’ p<0.05.

In *Drosophila* and other insects, one function of a local gut immune response is to prevent or minimize systemic immune induction arising from an oral infection. We investigated whether the peritrophic matrix plays a role in preventing the induction of a systemic response to microbiota growth within the gut following a blood meal. For each pool of dissected midguts we collected the associated carcass samples, consisting of the abdominal cuticle with the fat body attached, after removal of all other organs, namely the gut, ovaries and malpighian tubules. At 72h post blood feeding, we observed that the gut microbiota induced considerable systemic induction of *CEC1* in a subset of the polyoxin D-fed mosquitoes (Fig 5A), with *GAM1* expression exhibiting a similar trend (S5A Fig). The same effect was also observed in *APER1* knock down mosquitoes compared to the *LACZ* double stranded RNA-injected control, though in this case at 24h after the blood meal, where a significant increase in the expression of both *GAM1* and *LYSC1* was observed (S5B Fig). Again, this systemic immune response was fully dependent on the presence of the microbiota (S5B Fig).

Mechanistically, we considered that this could occur either via translocation of live bacteria from the gut to the hemocoel, or via bacteria or mosquito-derived molecules signaling from the gut to the hemocoel. We focused our analysis on peritrophic matrix disruption by polyoxin D feeding, as the *APER1* knock down cohort had sustained damage to the carcass during the injection process, providing a possible route of entry for exogenous bacteria. To investigate the former scenario, we attempted to amplify *16S rRNA* from the carcass samples of the control and polyoxin D fed cohorts (Fig 5B). In the antibiotic-treated mosquitoes, *16S rRNA* amplification was insignificant, at the level of the qRT-PCR negative controls, whereas *16S rRNA* was amplified above this level in a subset of both the non-antibiotic-treated control and polyoxin D-fed samples. We observed no significant difference in the relative total *16S rRNA* that was amplified from the carcasses of the control or polyoxin D-fed mosquito cohorts at 72h post blood feeding (Fig 5B).

Given that we observed an increase in the *Enterobacteriaceae* load in the gut at 24h post blood feeding in the polyoxin D fed cohort, and no difference in the overall bacterial load detected in the control and peritrophic matrix disrupted carcasses, we hypothesized that the systemic immune induction could be due specifically to translocation of this bacterial taxon. Indeed, at 72h post blood feeding we found a significant increase in the *Enterobacteriaceae* load in the peritophic matrix disrupted cohorts, with this family only being confidently detected in polyoxin D-fed pools of mosquito carcasses and not in the control pools (Fig 5C). No significant difference was observed in the incidence or load of the *Flavobacteriaceae* or *Acetobacteraceae* (S6C Fig). Furthermore, in the polyoxin D-fed group, *Enterobacteriaceae* detection in the carcass correlated significantly with *CEC1* induction, which was not the case in the control group (Fig 5D). *Flavobacteriaceae* and *Acetobacteraceae* abundance did not correlate with *CEC1* expression (S5C Fig). The clear relationship between *Enterobacteriaceae* load and *CEC1* expression, together with the fact that this family of bacteria is detected only in the carcasses of polyoxin D-fed mosquitoes, strongly suggests that this family of bacteria is able to translocate from the gut to the hemocoel upon disruption of the peritrophic matrix, seeding a systemic infection.

## Discussion

The data presented here reveal a complex and dynamic relationship between the midgut microbiota, the type I peritrophic matrix and local and systemic immune responses in adult female mosquitoes. In mammals, the inner and outer mucus layers of the gastrointestinal tract are composed of mucin glycoproteins and form a physical and biochemical barrier between the gut flora and the epithelial cells [38]. The mucus layer is at its thickest in the distal colon, the region of highest bacterial colonization, where it functions as a scaffold for AMPs and immunoglobulin A, and acts to protect against microbiota contact with the epithelium [39,40]. The outer mucus layer is known to interact with intestinal microbes [41], providing a habitat for O-glycan foraging taxa [42]. Thus, the defensive nature of mucus is dependent on the entrapment of microbes in the outer layer, but this equally facilitates microbial colonization in acting as a source of nutrition. The mosquito peritrophic matrix is structurally analogous to the vertebrate mucus layer, containing heavily glycosylated proteins, though in this case cross-linking chitin. Here, we reveal additional functional analogies between these two evolutionarily diverse biological structures in limiting gut microbiota growth and precluding bacterial invasion of the intestinal epithelia.

The type I peritrophic matrix is specifically produced by adult female midgut cells upon blood feeding and physically surrounds the blood bolus where blood digestion takes place. We show that synthesis of a structurally complete type I peritrophic matrix is dependent on the presence of the gut bacteria. Similar observations have been reported in other insects. In adult *Drosophila*, oral infection by *Erwinia carotovora carotovora* (*Ecc15*) has been shown to induce the expression of genes encoding proteins with CBDs, with the induction of a proportion of these genes being dependent on the Imd pathway, one of the main immune signalling pathways in the fly gut [18]. Similarly, a protein constituent of the adult *Drosophila* peritrophic matrix, Drosocrystallin, is induced by oral bacterial infection [19]. The presence of the gut microbiota in the tick *I*. *scapularis* has also been shown to be necessary for production of a peritrophic matrix of proper thickness, with this being dependent on STAT signalling [17]. Finally, a proteomic analysis of the type II peritrophic matrix of the tsetse fly identified 27 proteins derived from the secondary endosymbiont *Sodalis glossinidius*, suggesting similar interaction of this bacterium with the peritrophic matrix [43].

In the mosquito gut, the microbiota is also known to induce the Imd pathway [1,2]. The JAK/STAT pathway, which is activated in response to viral infections [44,45], is known to be responsive to bacterial infection [46] but has not yet been characterized as being microbiota responsive. It remains unclear which signalling pathway(s) are responsible for the microbiota-dependent induction of the *A*. *coluzzii* peritrophic matrix, though it is noteworthy that a number of regulated genes have canonical STAT binding sites in their upstream regions (S4 Table), indicating a potential role for the JAK/STAT pathway. Importantly, we observed significant microbiota-dependent regulation of the FoxO signalling pathway, which has recently been shown to facilitate bacteria-dependent synthesis of AMPs in *Drosophila* enterocytes [47] and may therefore also be considered a candidate pathway for peritrophic matrix induction.

Disruption of peritrophic matrix synthesis resulted in elevated load of the *Enterobacteriaceae* family of bacteria. Given that we observed bacteria co-locating with the peritrophic matrix, it is likely that the peritrophic matrix has antibacterial properties, whether by direct interaction or by sequestration within a hostile niche. Indeed, there is evidence that peritrophins from other organisms are able to interact directly with bacteria and have antibacterial functions [48,49], while a properly structured peritrophic matrix can also be a scaffold maintaining AMPs and other immune factors in the gut [38,50]. Interestingly, we found that AMP expression in the midgut correlated with the load of the *Enterobacteriaceae* and the *Acetobacteraceae* families, but not with the *Flavobacteriaceae*. This could suggest that the *Flavobacteriaceae* occupy niches within the gut that are not surveyed by the immune system, whilst the *Enterobacteriaceae* and *Acetobacteraceae* live more proximally to the epithelium, likely within or upon the peritrophic matrix. It remains unclear why the load of the *Enterobacteriaceae* but not the *Acetobacteraceae* is limited by the presence of the peritrophic matrix after blood feeding. This observation is, however, consistent with a previous study that found *Asaia*, a major constituent of the *Acetobacteraceae* family in our mosquito colony, to be resistant to the mosquito immune response [51].

Intriguingly, we observed bacteria present throughout the ectoperitrophic space, including proximally to the epithelium. This could suggest that the bacteria that are directly associated with the peritrophic matrix are efficiently excreted with the blood bolus, whilst those located in the ectoperitrophic space remain in the gut, seeding the bacterial population in the next gonotrophic cycle.

In addition to local effects in the gut, we observe the induction of a systemic immune response upon disruption of the peritrophic matrix and show that this is associated with the break down in the integrity of the gut barrier and translocation of bacteria of the *Enterobacteriaceae* family into the body cavity. In humans, bacterial translocation is thought to be a common occurrence in healthy individuals and can present as a complication in the critically ill [52]. It is understood to be a consequence of bacterial overgrowth [53], disruption of the mucosal barrier [54] and impaired immune defense [55]. In *Drosophila*, it has recently been demonstrated that the enteric nervous system controls peritrophic matrix permeability, and that when permeability is compromised flies succumb to bacterial dissemination throughout the body following oral bacterial infection [56]. In concurrence with this, our data suggest that the peritrophic matrix is a key barrier to prevent or limit translocation of microbiota-derived *Enterobacteriaceae* into the mosquito body cavity.

In our *A*. *coluzzii* colony, abundant genera of the *Enterobacteriaceae* family include *Cedecea*, *Enterobacter*, *Ewingella* and *Serratia* [36]. Species of *Enterobacter* have been demonstrated to invade epithelial cells [57] and *Serratia marcescens* is a model pathogen in *Drosophila* that, when introduced orally, is able to traverse the gut epithelium [58]. More generally, *Enterobacteriaceae* have been associated with the induction of colitis, or inflammation of the colon, in mice [59]. Bacterial translocation has not been formally demonstrated in the mosquito, but certain lines of evidence point to this phenomenon. Importantly, a GFP-tagged *Asaia* strain is known to be able to colonize both the reproductive organs and salivary glands when fed to adults in a blood or sugar meal [60]. Furthermore, knock down of immune effectors can result in bacterial proliferation in the hemolymph even in the absence of infection, though in both of these cases the cuticle was damaged by RNAi injection, providing a possible route of entry for exogenous bacteria [61,62]. Taken with the results presented here, these data suggest that at least some native constituents of the mosquito gut microbiota are able to disseminate throughout the body, and that the peritrophic matrix plays a key role in limiting this dissemination.

## Materials and methods

### Mosquito colony maintenance

The *A*. *coluzzii* Ngousso colony was used in all experiments described here. Eggs were hatched in 0.1% salt water and larvae fed Tetramin or Nishikoi fish food. All adults were allowed *ad libitum* access to 5% w/v fructose solution and females were maintained on human blood. The insectary was maintained at 27°C (±1°C), 70–80% humidity with a 12h light/dark cycle.

### Human blood feeding

Human blood for mosquito feeding was acquired from the NHS blood service. During feeding, blood was maintained at 37°C on a membrane-feeding device or in a parafilm-covered Petri dish warmed with a handwarmer. Mosquitoes were allowed to feed for 1h and non-engorged mosquitoes were removed within 24h. Mosquitoes were offered egg dishes for oviposition the night before each subsequent blood meal. Antibiotics (60U/ml penicillin, 60μg/ml streptomycin and 50μg/ml gentamicin) or an equal volume of water were supplemented in the sugar solution offered from emergence, in the blood meal and in the egg dish provided for oviposition. For polyoxin D feeding, 0.01M stock solution was prepared from powder in water and added to human blood at a final concentration of 100μM immediately before feeding. An equal volume of water was added to the control blood meal.

### Double stranded RNA preparation and injection

Double stranded RNA (dsRNA) was used for transient *in vivo* knock down of target genes by RNAi. The target region was amplified from total *A*. *gambiae* cDNA using primers flanked with the T7 RNA polymerase promoter sequence (sequences are listed in S5 Table). dsRNA was synthesised from the PCR product by overnight incubation at 37°C with T7 polymerase and dNTPs from the MEGAscript RNAi kit, according to the manufacturer’s instructions. dsRNA was purified using the Qiagen RNeasy kit, adjusted to a concentration of 6000 ng/μl, and stored in aliquots at -20°C. 69 nl of 6000 ng/μl dsRNA (totalling 414 ng) was injected into the thorax of CO2- anaesthetised 0–2 day old female mosquitoes using the Nanoject II (Drummond Scientific). dsRNA against a region of the *lac* operon (LACZ), not present in the *A*. *gambiae* genome, was injected as a control for the injection process.

### Midgut and carcass dissection

Prior to dissection mosquitoes were ‘surface sterilized’ by immersion in 75% ethanol for 3–5 min and washed three times in phosphate buffered saline (PBS) to minimize environmental contamination from cuticle bacteria into dissected midgut samples. Midguts were removed under a dissecting microscope, frozen immediately on dry ice in pools of 20 (for RNA sequencing), 3–5 (for excretion experiment and *APER1* experiments) or 8–10 (for all other experiments), and stored at -20°C until processing. For carcass dissections, the abdominal carcass and attached fat body tissue was dissected, ensuring that all other organs (ovaries, gut, malpighian tubules) were removed. Carcasses were frozen immediately on dry ice in pools of 3–5 (for *APER1* experiments) or 8–10 (for polyoxin D experiments) then stored at -20°C until processing.

### RNA extraction and cDNA synthesis

Frozen tissues were homogenized in TRIzol (Invitrogen) and chloroform using a Precellys24 tissue homogenizer with bead beating (Bertin). RNA was precipitated from the aqueous phase with isopropanol, washed twice in 70% ethanol and resuspended in water. For RNA sequencing experiments, samples underwent a further column purification using the Qiagen RNeasy kit. For qRT-PCR experiments, cDNA was synthesized from up to 500 ng RNA using the Takara reverse transcriptase kit, according to the manufacturer’s instructions.

### RNAseq library preparation and sequencing

Libraries for sequencing were prepared in accordance with the Illumina TruSeq RNA sample preparation v2 guide (Part # 15026495, rev.D, September 2012) for Illumina Paired-End Indexed Sequencing. PolyA mRNA first underwent two rounds of purification using Illumina poly-T oligo-attached magnetic beads. During the second elution, the polyA mRNA was fragmented and primed with random hexamers for cDNA synthesis. After first strand cDNA synthesis, the RNA template was removed and a replacement strand was synthesized to generate double stranded cDNA. Ends were then repaired, dA base added and Illumina indexing adapters ligated. cDNA fragments with adapters on both ends underwent 15 cycles of PCR. Libraries were validated with the Agilent 2100 bioanalyzer to check size distribution. Samples were quantified by qRT-PCR, the concentrations normalized and samples pooled according to biological replicate. Pools were loaded at 10 pM onto four lanes of an Illumina flowcell v3 and sequenced using the Illumina HiSeq 1500, 2 X 100 bp paired-end run. Sequences are deposited in the NCBI Sequence Read Archive under the BioProject ID PRJNA385903.

### RNAseq quality control, read filtering and sequence alignment

Quality control, filtering and alignment were conducted in the Galaxy platform [63–65]. Groomed FASTQ files underwent adapter clipping (ILLUMINACLIP with Truseq3 adapter sequences) and were then trimmed by sliding window, averaging a minimum Phred quality score of 20 over 4 bases (Trimmomatic tool version 0.32.1). Only reads with both mate pairs being longer than 20 bp were processed further. These were aligned by Bowtie2 (version 0.4) to a custom built index to filter out non-mRNA reads, composed of all sequences annotated as *A*. *gambiae* in the SILVA rRNA database (release 119) [66], plus all sequences annotated as *A*. *gambiae* tRNAs and mitochondrial rRNAs in the AgamP4.2 geneset in Vectorbase. The splice aware aligner Tophat2 v2.0.9 was used to align paired-end reads to the *A*. *gambiae* PEST genome AgamP4. The mean inner distance between mate pairs was set to -25 with a standard deviation of 60. Default settings were used for alignment with the following exceptions: the maximum number of mismatches allowed between a read and the reference sequence was 5 to allow for the highly polymorphic nature of the *A*. *gambiae* genome, and the minimum intron length was set to 30 bp. The accepted hits were filtered such that only reads that were uniquely mapped were accepted for downstream analysis.

### Generation of count data and differential expression calling

Aligned reads were converted to gene count data using HTSeq, specifying the union mode [67]. The input gtf file was AgamP4.2 after removal of all features annotated as rRNA. Differential expression analysis was conducted with the DESeq2 package [68], using HTSeq count tables as input files. For each gene, the DESeq2 package fits a generalized linear model (GLM) with a negative binomial distribution. For pairwise comparisons at each time point the input parameters were “replicate” and “treatment” (i.e., plus/minus antibiotics), and “treatment” was removed in the reduced model. DESeq2 applies the Wald test to assess statistical significance followed by the Benjamini-Hochberg adjustment for multiple testing. Genes with adjusted p-values <0.1 were considered significantly differentially expressed.

### Clustering analysis

Variance stabilizing transformation of count data was performed in DESeq2 prior to clustering. A median-transformed value of the four replicates was calculated for each condition and soft clustering performed in Mfuzz [69]. Soft clustering does not require *a priori* gene filtering, is noise robust and allows genes to be placed in more than one cluster, making it ideal for time-course data. The fuzzifier *m* was chosen with the *mestimate* function, and the optimal number of 12 clusters was selected based on when the minimum distance between cluster centroids (*Dmin*) declines at a reduced rate.

### KEGG/GO enrichment analysis

KEGG and GO term enrichment analysis for differentially expressed genes were performed in g:Profiler [70]. Genes were ordered by their fold change for input to the software and a Bonferroni adjustment was made for multiple testing.

### qRT-PCR

qRT-PCR was used to quantify *A*. *gambiae* mRNA levels, as well as bacterial load by amplification of the 16S rRNA gene, employing primers that anneal to a region of the sequence that is common to all eubacteria or specific to bacterial families examined. Primer sequences are listed in S5 Table. In each case, the *A*. *coluzzii* ribosomal protein encoding gene S7 (AGAP010592) was used as an internal control of the quantity of input RNA. Expression ratios were calculated using primer efficiencies that were determined by amplification of serially diluted targets. qRT-PCR amplifications were performed in duplicate using the SYBR premix ex Taq kit (Takara) in a total volume of 10μl on a 7500 Fast Real Time PCR machine (Applied Biosystems).

### Histological analysis

For hematoxylin and eosin (H&E) staining, whole mosquito abdomens were dissected 24h post blood feeding, fixed overnight at 4°C in Duboscq’s-Brasil fixative (0.4% w/v picric acid, 53% ethanol, 27% formalin, 7% glacial acetic acid) and washed in 70% ethanol. Abdomens were then processed to paraffin wax and sections cut to 4μm onto Superfrost Plus slides (VWR). For Gram staining, immunostaining and calcofluor white staining, whole mosquito abdomens were dissected 24h post blood feeding, fixed overnight at 4°C in 4% formalin, washed in *Aedes* saline (150mM sodium chloride, 1.4mM calcium chloride, 2mM potassium chloride, 1.2mM sodium hydrogen carbonate, pH 7.2), and embedded and sectioned as described. For Gram staining, sections were dewaxed and stained according to the Gram/Twort protocol. Slides were observed under a Leica DMR microscope. For immunostaining, sections were stained with *E*. *coli* polyclonal antibody at 1:400 (bs-2351R Bioss Antibodies) and a goat anti-rabbit antibody bound to Alexa 647 (Thermofisher A21245) was used as secondary (1:1000). Slides were mounted in Prolong Gold antipode (Invitrogen) and observed under a Zeiss Widefield Axio Observer Microscope. For calcofluor white staining, sections were dewaxed and rinsed in distilled water before being stained for 2h in calcofluor white solution in the dark (Sigma 18909). Sections were observed under a Zeiss Widefield Axio Observer Microscope.

### Statistical analysis

qRT-PCR data (including gene expression and 16S analyses) were analyzed by generalized linear mixed models (GLMMs) in R (version 3.1.2). GLMMs fit both fixed-effect parameters and random effects in a linear predictor via maximum likelihood. Mixed effect models were used to account for the use of multiple sample pools per condition within each independent replicate, avoiding issues of pseudoreplication. Statistical significance was assessed by an ANOVA test on a linear mixed effect regression model (lmer, in the lme4 package). Correlation was analyzed by a Spearman rank-order correlation test.

## Supporting information

S1 Table GO biological processes and KEGG terms that are significantly enriched after antibiotic treatment. Terms were identified in g:Profiler. Shaded entries indicate terms that are enriched in genes downregulated by antibiotic treatment. Non-shaded entries indicate terms that are enriched in genes upregulated by antibiotic treatment. Only terms with two or more genes are listed. Statistical significance was assessed with a Fisher’s one tail test with Bonferroni correction for multiple testing. ‘n’ indicates the number of genes associated with that term that are significantly regulated at the time point indicated.
(DOCX)

S2 Table Correspondence between microbiota-regulated genes and constituents of the peritrophic matrix. Among genes identified in (Dinglasan et al., 2009) as top candidates for peritrophic matrix components, entries highlighted in grey are identified in this study as being transcriptionally regulated by the presence of the microbiota. Abundance ranking refers to the relative abundance of the protein in the mass spectrometric analysis of Dinglasan et al. Fold change given in the final column is the transcriptional log2 fold change identified here upon antibiotic treatment. CBD = chitin binding domain.
(DOCX)

S3 Table Regulation of immune effector encoding genes in the midgut by antibiotic treatment. Log2 fold changes of immune effector encoding genes (AMPs and C-type lysozymes) upon antibiotic treatment. Highlighted entries indicate statistical significance (adjusted p-value < 0.1, Wald test).
(DOCX)

S4 Table 500 bp 5’ of CDS start sites of genes encoding putative peritrophic matrix components. Sequences highlighted in red are consensus STAT binding motifs (TTCNNN(N)GAA). Sequences were taken from Vectorbase (AgamP4.5 gene set).
(DOCX)

S5 Table Primer sequences used for qRT-PCR analysis and dsRNA synthesis. (DOCX)

S1 Fig Soft clustering analysis of midgut gene expression in control and antibiotic time courses. Clusters were generated in the Mfuzz package. Each line represents one gene in the cluster, with line colour indicating strength of membership in the cluster (red being the strongest membership and green the weakest). C = control, Ab = antibiotics.
(TIF)

S2 Fig Feeding with 100μM polyoxin D compromises the integrity of the peritrophic matrix. H&E **(A)** and calcofluor white **(B)** stained thin sections of engorged midguts 24 h post blood feeding with the addition of 100μM polyoxin D to the blood meal. In **(B)**, arrowheads indicate staining of the cuticle (grey arrows) and the fragmentary peritrophic matrix (white arrows).
(TIF)

S3 Fig The effect of peritrophic matrix disruption by polyoxin D feeding and *APER1* knock down on immune induction and bacterial load in the midgut 24h post blood feeding. **(A)** *CEC1* expression, relative to *AgS7*, in the midgut 24h after feeding with a blood meal supplemented with 100μM polyoxin D or an equal volume of water (control), plus or minus antibiotic treatment, as determined by qRT-PCR. **(B)** *CEC1* and *GAM1* expression in the midgut of APER1 and LACZ (control) knock down mosquitoes 24h after blood feeding, plus or minus antibiotic treatment, as determined by qRT-PCR. **(C)** Total bacteria load, *Flavobacteriaceae* load, and *Acetobacteraceae* load 24h after feeding with 100μM polyoxin D or a control blood meal, relative to *AgS7*, as determined by qRT-PCR with family specific or universal 16S primers. **(D)** *Enterobacteraceae* load in APER1 or LACZ (control) knock down mosquitoes 24h after blood feeding. A-D: Each dot represents a pool of 8–10 (polyoxin D experiments) or 3–5 (APER1 experiments) guts, derived from 4–5 independent experiments. Ratios are normalized within biological replicates to the mean of the control pools. Mean plus/minus standard error is indicated. Statistical significance was assessed by an ANOVA on a linear mixed effect regression model. ‘*’ p<0.05.
(TIF)

S4 Fig The effect of peritrophic matrix disruption on total bacteria load and *Flavobacteriaceae* load in the midgut 72h post blood feeding. Total bacteria load and *Flavobacteriaceae* load in the midgut 72 h after feeding with a blood meal supplemented with 100μM polyoxin D or an equal volume of water (control), as determined by qRT-PCR with universal of family-specific 16S primers. Each dot represents a pool of 8–10 guts, derived from 4 independent experiments. Ratios are normalized within biological replicates to the mean of the control pools. Mean plus/minus standard error is indicated.
(TIF)

S5 Fig The effect of peritrophic matrix disruption on systemic immune induction. **(A)** *GAM1* expression in the carcass 72 h after feeding with a blood meal supplemented with 100μM polyoxin D or water as a control, plus or minus antibiotic treatment, as determined by qRT-PCR. **(B)** *GAM1* and *LYSC1* expression in the carcass of APER1 and LACZ (control) knock down mosquitoes, 24 h after a human blood meal. A-B: Each dot represents a pool of 8–10 (A) or 3–5 (B) carcasses, derived from 4 independent experiments. Ratios are normalized within biological replicates to the mean of the control pools. Mean plus/minus standard error is indicated.
(TIF)

S6 Fig The effect of peritrophic matrix disruption on bacteria detection in the carcass. **(A)** *Flavobacteriaceae* and *Acetobacteraceae* load in the carcass 72h after feeding with a blood meal supplemented with 100μM polyoxin D or water as a control. **(B)** Scatter plots of relative *Flavobacteriaceae* and *Acetobacteraceae* load against *CEC1* expression in the carcass of polyoxin D fed mosquitoes at 72h post blood feeding. A-B: Ratios are normalized within each biological replicate to the highest value across all conditions (‘100%’). Each dot represents a pool of 8–10 (polyoxin D carcasses, derived from 4 independent experiments. In A, the mean plus/minus standard error is indicated. In B, Spearman’s rank correlation coefficient and associated p-values are indicated.
(TIF)

S1 File Output of DESeq2 differential expression analysis. Tab 1 (‘Significant genes’) lists all genes that were found to be differentially expressed in antibiotic treated versus control guts at one or more time points, together with their adjusted p values at each time point. Subsequent tabs contain the full output of DESeq2 differential expression analysis at each time point.
(XLSX)
