# Supplementary material for: Microbiota-induced peritrophic matrix regulates midgut homeostasis and prevents systemic infection of malaria vector mosquitoes
Source: PLoS Pathog. 2017 May 17;13(5):e1006391. doi: 10.1371/journal.ppat.1006391 (PMC5448818; doi:10.1371/journal.ppat.1006391)
Supplement: S1 Table — Terms were identified in g:Profiler. Shaded entries indicate terms that are enriched in genes downregulated by antibiotic treatment. Non-shaded entries indicate terms that are enriched in genes upregulated by antibiotic treatment. Only terms with two or more genes are listed. Statistical significance was assessed with a Fisher’s one tail test with Bonferroni correction for multiple testing. ‘n’ indicates the number of genes associated with that term that are significantly regulated at the time point indicated. (DOCX) [file ppat.1006391.s001.docx]

| **S1 Table. GO Biological Processes and KEGG terms that are significantly enriched after antibiotic treatment** | | | |
| --- | --- | --- | --- |
| **Time point** | **Term description** | **Term ID** | **Adjusted p value** |
| 0h | Defense response to bacterium (n=4) | GO:0042742 | 0.00000365 |
|  | Peptidoglycan catabolic process (n=3) | GO:0009253 | 0.00448 |
|  | Neurotransmitter transport (n=2) | GO:0006836 | 0.00642 |
|  | Glycosaminoglycan degradation (n=2) | KEGG:00531 | 0.00816 |
|  | Oxidation-reduction process (n=16) | GO:0055114 | 0.00072 |
|  | FoxO signaling pathway (n=2) | KEGG:04068 | 0.05 |
| 5h | Proteolysis (n=39) | GO:0006508 | 0.00834 |
|  | Single organism carbohydrate metabolic process (n=64) | GO:0044710 | 0.0000171 |
|  | Signal peptide processing (n=4) | GO:0006465 | 0.000992 |
|  | Organophosphate catabolic process (n=6) | GO:0046434 | 0.0421 |
|  | Histidine metabolism (n=4) | KEGG:00340 | 0.0216 |
|  | beta-Alanine metabolism (n=5) | KEGG:00410 | 0.0221 |
|  | N-Glycan biosynthesis (n=13) | KEGG:00510 | 0.000000159 |
|  | Protein export (n=8) | KEGG:03060 | 0.000822 |
|  | Protein processing in endoplasmic reticulum (n=25) | KEGG:04141 | 3.03E-09 |
|  | Ascorbate and aldarate metabolism (n=5) | KEGG:00053 | 0.000169 |
|  | Ribosome biogenesis (n=9) | GO:0042254 | 0.000000187 |
|  | Ribosome biogenesis in eukaryotes (n=14) | KEGG:03008 | 2.10E-12 |
|  | Glutathione metabolism (n=4) | KEGG:00480 | 0.0147 |
| 24h | Defense response to bacterium (n=4) | GO:0042742 | 0.0000549 |
|  | Peptidoglycan catabolic process (n=3) | GO:0009253 | 0.0000378 |
|  | Folate biosynthesis (n=2) | KEGG:00790 | 0.05 |
| 72h | Sphingolipid metabolic process (n=4) | GO:0006665 | 0.0337 |
|  | Defense response to bacterium (n=4) | GO:0042742 | 0.00000179 |
|  | Protein processing in endoplasmic reticulum (n=14) | KEGG:04141 | 0.000114 |
|  | Ribosome biogenesis (n=7) | GO:0042254 | 0.0422 |
|  | ncRNA processing (n=8) | GO:0034470 | 0.00752 |
|  | Organonitrogen compound biosynthetic process (n=22) | GO:1901566 | 0.0222 |
|  | Oxidation-reduction process (n=26) | GO:0055114 | 0.0219 |
|  | Ubiquinone biosynthetic process (n=3) | GO:0006744 | 0.0211 |
|  | Cofactor biosynthetic process (n=6) | GO:0051188 | 0.0374 |
|  | Ribosome biogenesis in eukaryotes (n=16) | KEGG:03008 | 6.62E-10 |
| 96h | Defense response to bacterium (n=3) | GO:0042742 | 0.00024 |
|  | Peptidoglycan catabolic process (n=3) | GO:0009253 | 0.000139 |
|  | Porphyrin and chlorophyll metabolism (n=2) | KEGG:00860 | 0.0049 |
|  | Starch and sucrose metabolism (n=2) | KEGG:00500 | 0.00124 |
| Terms were identified in g:Profiler. Shaded entries indicate terms that are enriched in genes downregulated by antibiotic treatment. Non-shaded entries indicate terms that are enriched in genes upregulated by antibiotic treatment. Only terms with two or more genes are listed. Statistical significance was assessed with a Fisher’s one tail test with Bonferroni correction for multiple testing. ‘n’ indicates the number of genes associated with that term that are significantly regulated at the time point indicated. | | | |
