# Supplementary material for: Microbiota-induced peritrophic matrix regulates midgut homeostasis and prevents systemic infection of malaria vector mosquitoes
Source: PLoS Pathog. 2017 May 17;13(5):e1006391. doi: 10.1371/journal.ppat.1006391 (PMC5448818; doi:10.1371/journal.ppat.1006391)
Supplement: S2 Table — Among genes identified in (Dinglasan et al., 2009) as top candidates for peritrophic matrix components, entries highlighted in grey are identified in this study as being transcriptionally regulated by the presence of the microbiota. Abundance ranking refers to the relative abundance of the protein in the mass spectrometric analysis of Dinglasan et al. Fold change given in the final column is the transcriptional log2 fold change identified here upon antibiotic treatment. CBD = chitin binding domain. (DOCX) [file ppat.1006391.s002.docx]

| S2 Table. Genes identified in Dinglasan et al., 2009 as top candidates for protein constituents of the peritrophic matrix | | | | |
| --- | --- | --- | --- | --- |
| Gene ID | **Gene Name** | **Relative abundance (n=209)** | **Description** | **Log2 fold change (time point) upon antibiotic treatment** |
| AGAP006795 | AgAper1 | 7 | 2 CBD | -1.21 (24h); -1.15 (96h) |
| AGAP006796 | AgAper9 | 19 | 1 CBD | -0.74 (5h); -1.14 (24h); -.1.74 (96h) |
| AGAP009830 | AgAper14 | 18 | 1 CBD |  |
| AGAP010364 | AgAper25a | 22 | 2 CBD |  |
| AGAP001819 | AgAper25b | 24 | 1 CBD | -1.09 (96h) |
| AGAP011615 | AgAper26 | 21 | 3 CBD |  |
| AGAP010363 | AgAper29 | 25 | 2 CBD |  |
| AGAP011616 | AgAper30 | 20 | 3 CBD |  |
| AGAP006433 | AgAper34 | 26 | 4 CBD | -0.63 (5h) |
| AGAP006434 | AgAper57 | 23 | 4 CBD | -0.54 (5h) |
| AGAP006432 | AgICHIT | 17 | 2 CBD | -1.04 (5h); -1.63 (24h); -0.79 (72h) |
| AGAP006414 | AgChitinase | 5 | 1 CBD; Chitinase |  |
| AGAP006442 |  | 31 | Conserved hypothetical protein |  |
| AGAP004883 |  | 27 | Conserved hypothetical protein |  |
| AGAP007860 |  | 33 | Conserved hypothetical protein |  |
| AGAP007612 |  | 28 | Conserved hypothetical protein | -0.46 (72h) |
| AGAP002851 |  | 32 | Conserved hypothetical protein | 0.91 (24h) |
| AGAP001352 |  | 30 | Conserved hypothetical protein |  |
| AGAP010132 | AgSCRBQ1 | 34 | Conserved hypothetical protein |  |
| AGAP006398 |  | 29 | Conserved hypothetical protein |  |
| AGAP000570 |  | 2 | Conserved hypothetical protein | -1.19 (24h); -1.46 (96h) |
| AGAP007745 |  | 4 | Conserved hypothetical protein | -0.67 (5h); -2.56 (24h); -0.72 (72h); -2.15 (96h) |
| AGAP006194 |  | 10 | Conserved hypothetical protein | -1.14 (0h); -0.79 (5h); -1.40 (24h); -1.26 (72h); -1.27 (96h) |
| AGAP009313 | AgPM9 | 6 | Conserved hypothetical protein | -0.98 (0h); -0.58 (5h); -1.09 (24h); -1.41 (72h); -1.24 (96h) |
| All entries highlighted in grey are identified in this study as being transcriptionally regulated by the presence of the microbiota. Abundance ranking refers to the relative abundance of the protein in the mass spectrometric analysis of Dinglasan et al. Fold change given in the final column is the transcriptional log2 fold change identified here upon antibiotic treatment. CBD= chitin binding domain. | | | | |
