# Supplementary material for: Microbiota-induced peritrophic matrix regulates midgut homeostasis and prevents systemic infection of malaria vector mosquitoes
Source: PLoS Pathog. 2017 May 17;13(5):e1006391. doi: 10.1371/journal.ppat.1006391 (PMC5448818; doi:10.1371/journal.ppat.1006391)
Supplement: S3 Table — Log2 fold changes of immune effector encoding genes (AMPs and C-type lysozymes) upon antibiotic treatment. Highlighted entries indicate statistical significance (adjusted p-value < 0.1, Wald test). (DOCX) [file ppat.1006391.s003.docx]

| **S3 Table.** AMP genes that are significantly regulated in the midgut by antibiotic treatment. | | | | | | |
| --- | --- | --- | --- | --- | --- | --- |
| **Gene name** | **Gene ID** | **0h** | **5h** | **24h** | **72h** | **96h** |
| *CEC1* | AGAP000693 | -1.72 | -1.25 | -1.86 | -1.69 | -1.80 |
| *CEC2* | AGAP000692 | -0.28 | -0.04 | 0.09 | -0.66 | -0.74 |
| *CEC3* | AGAP000694 | -1.24 | -0.73 | -1.29 | -1.84 | -1.50 |
| *DEF1* | AGAP011294 | -0.93 | -0.46 | -0.80 | -1.05 | -1.19 |
| *GAM1* | AGAP008645 | -1.45 | -1.73 | -2.85 | -1.74 | -2.01 |
| *LYSC1* | AGAP007347 | -2.00 | -1.09 | -1.64 | -2.02 | -0.57 |
| *LYSC7* | AGAP007386 | -0.32 | 0.12 | 0.18 | -0.80 | -0.71 |
| Log2 fold changes of immune effector encoding genes (AMPs and C-type lysozymes) upon antibiotic treatment. Highlighted entries indicate statistical significance (adjusted p-value < 0.1, Wald test). | | | | | | |
