# Supplementary material for: Microbiota-induced peritrophic matrix regulates midgut homeostasis and prevents systemic infection of malaria vector mosquitoes
Source: PLoS Pathog. 2017 May 17;13(5):e1006391. doi: 10.1371/journal.ppat.1006391 (PMC5448818; doi:10.1371/journal.ppat.1006391)
Supplement: S4 Table — Sequences highlighted in red are consensus STAT binding motifs (TTCNNN(N)GAA). Sequences were taken from Vectorbase (AgamP4.5 gene set). (DOCX) [file ppat.1006391.s004.docx]

| Gene ID | Gene name | Sequence (500bp 5’ of CDS start site) |
| --- | --- | --- |
| AGAP002851 | - | TATATCATTCTCAATGTTGTGAACTTGAGCCTATTCGCTTTTCGCTTATTTAATAAGCCG  TAATGAAACAATTATTCTACGCCACGA**TTCTAGTGAA**ATTCGATTGCAAGATATAGATTA  AGTAGAAGTTCAGAAATACTCCCAAAATGTGCACATTCTTAAAAGATGCTTACACTTATT  GTACAAATATTACTGTGAAATACTCAATTAAACACCTTTTATTAGAAAACACAAACAAAC  TGATTATCCCTTATACCAAACCCCCTAAACAATGAGTGCATTGTCCCCACACTATCTTTC  ATTCCACTTGAAGCAACCAGATG**TTCACCGAA**CAGGACGGTAACTAATCCATGCCTATCG  AGATGTAACTAGTCAATCCTCTCGAGCCGTGTAACACATGCTTGTAGGATGAAGCTTGAT  TGAATAGCTTTCTTCCATTATTTAAGCAAGTGCGCCAGTGTATCTTCTCACCATTCCGTA  CCATCACATTCTGACGCAAG |
| AGAP000570 | - | AAAGTTTCTTCCCACTTTCTAGGCTCTAGGCACCGGCGTAGAAGACATCCGGCGCACTCC  AACCGGCACTGGCGATGAAAGGGATGGCGTTGTGGCGATGTTAAGTGTCCAATAAAGAGG  ATGATACCTCTCTCACT**TTCAACCGAA**ACACTTTACGCTGAAGGGCGCAAAGGCTTCGAG  CGATCGAATTGATTGACTTCCTATTCCAGGGATTACGGGCGCGAAGATATCACAGCGGGT  TTGATTGGGCAGGTGACTCAATCTCAACGTCCCATCGCGTGGACAGGTACGGGGATGTTT  GTTGTTGTTGTTGTTTATGCAGTTCGTACCGGCTCTCGTGGCGATAAGATGCCGACGCGA  TTCCCTCTGCGAGTGCGCTATCTTTCGAGTGGCATTTCCGTTTGCACTAACCACTAACCT  AGTTCTACTGCCGTGTATAAAAGGTGCTTCCGAACGTCTGGCCGCCATTGTATTGCAGCT  CTCCTCCGCACAAATCAACC |
| AGAP001205 | Chitin synthase | AAACTCATACCGGTCCTAGCCAAGCCTTGTTTTTTACTATCGAAAACAAAACGGTGGTCA  TTCGATCAGGCCGCGCCTTCGCATCCTTGCACTCAGGATACGCACGACGGAATTGGTGCG  TGTTTGTTTACCCAATTGATTGAATTTCGATTACCGTTAGCGGCTGTGAGTGAATGAGTG  AG**TTCCCAGGAA**AGGGGTTTTGTGTGCACGTGTGCTCAAAGGTCTGTGCCGCAAATCCTA  GGCACAGTCTCCCCCGCAGAGGAAAACGCTGTACGATCCGAGTGTAATGTGATTATCGAT  CCGATCGTGTCGTGTCGTGGGGCAGGTGTCGAAATTGCCCTCGTATCATTAAACTCCACT  TCCGGGGGAAGGTGCGGGTGAACAGTGAATGTGATAACAAAAAAGCACCCAACTCACACA  CACAGTGCCAGTGCACAGTTATTGAAAAGCGATTGCATCACCGCGAGGGCATCCGTGGAG  CTCGGGCTCCAAAGTGCAGG |
| AGAP006194 | - | CAGAACACCACACGTAAGGTTCTTCGTCACCTGATCCACTCACGAAAGAAGCAGAGCTTG  GATAATACTTGCAACAGCTTAGCAGCTTAAAATCGTGTTGCTACTTCCGTCAAACATGAT  TTCCGGCAAGATCTGGTTAACGGAAAGATAAACTGTTATTTAACAAAAGCAAAGAAACTA  AATG**TTCTTAGAA**TCGTACTAGACTTGTAGATAAGAATGAAATACTAATATTGAAATATC  TAACATTAAACAAGGCAAACGTTAAAGATGTACACAGACATGATAGTAATATATATTGTT  GTTCATACGCCTGCAAATCAAATGTTATCACTCACTTGTTGTCTCTTGTATCATCAGCCA  AACGATGCTAAGATAAAGTGAAATGGTATTTGTACGTCTGAAAAACCCCAGAACCTGCGC  AAAGATAAAACTATTTATAATGCATCGCACTCTGAATTAAATTGCAGTTCCGTGCGGTGC  ATCACACAGCAGGAAGTAAA |
| AGAP006795 | APER1 | ACCATAAAATCCCGTCACAGCGGTGGATGGAGTTGGCGGTGATATAAAATCGAACCACCC  GGTTGTCGGTTGTGATCGTTGGACCGAGAATGCGTCACGTGCGTTGGGGTTTGTGTGATA  TTATGCACTATGGGTTTCATTAGTGTGGACGCGGGAACATTTGATTTTTTTTATTTTTAA  TGTAATATTTAATTGCATTAATCCAGAGCTTTGTGATAACGTCACACTTTCTCACGAGCG  GTCAGGGTGTGCAACTTAGTAGACTTCAAGTGTCAACGGCTTTGCTTATCCACAAGCATG  AATGAACGTCGGATTGCTAAAGTACACAGTTCTGAGAGGCTCGTCA**TTCGATTGAA**ATGT  GTCCAGCATCACTCATCCACTTCAGATTATCCATTGTTATCATCGCACCGAAGCATCGCA  ATAAAAAGGACAGACTGCACTGGCCAGTGAGTGCAGTACGGAGTGAAACGCCTTTGCCAG  TACAATCTAACCGAAATAAA |

**S4 Table. 500 bp 5’ of CDS start sites of genes encoding putative peritrophic matrix components**

Sequences highlighted in red are consensus STAT binding motifs (TTCNNN(N)GAA). Sequences were taken from Vectorbase (AgamP4.5 gene set).
