# Supplementary material for: Microbiota-induced peritrophic matrix regulates midgut homeostasis and prevents systemic infection of malaria vector mosquitoes
Source: PLoS Pathog. 2017 May 17;13(5):e1006391. doi: 10.1371/journal.ppat.1006391 (PMC5448818; doi:10.1371/journal.ppat.1006391)
Supplement: S5 Table — (DOCX) [file ppat.1006391.s005.docx]

| **S5 Table. Oligonucleotide sequences** | | | |
| --- | --- | --- | --- |
| **Gene name** | **Gene ID** | **Forward primer** | **Reverse primer** |
| *S7* | AGAP010592 | GTGCGCGAGTTGGAGAAGA | ATCGGTTTGGGCAGAATGC |
| *Eubacteria 16S* | - | TCCTACGGGAGGCAGCAGT | GGACTACCAGGGTATCTAATCCTGTT |
| *CEC1* | AGAP000693 | ACCAACCAACCACCAAACAAC | CTTCTCTGCTGCCTTGAACACT |
| *GAM1* | AGAP008645 | CTATCTCAACCGGAAGGGCG | GCCAAACTTTCGCTTGCAGT |
| *Flavobacteriaceae 16S* | - | TAAGGTTGAAGTGGCTGGAATAA | GTCCATCAGCGTCAGTTAAGACT |
| *Enterobacteriaceae 16S* | - | CGTGCTACAATGGCATATACAAAGAGAAG | AGCATTCTGATCTACGATTACTAGCGATTC |
| *Acetobacteriaceae 16S* | - | GTGCCGATCTCTAAAAGCCGTCTCA | TTCGCTCACCGGCTTCGGGT |
| *APER1* (dsRNA synthesis) | AGAP006795 | taatacgactcactatagggagaATGGTGTACATTCCGCACGA | taatacgactcactatagggagaCACAGTGGCACACTTCAAGC |
