# Supplementary figures and images for: Microbiota-induced peritrophic matrix regulates midgut homeostasis and prevents systemic infection of malaria vector mosquitoes
Source: PLoS Pathog. 2017 May 17;13(5):e1006391. doi: 10.1371/journal.ppat.1006391 (PMC5448818; doi:10.1371/journal.ppat.1006391)

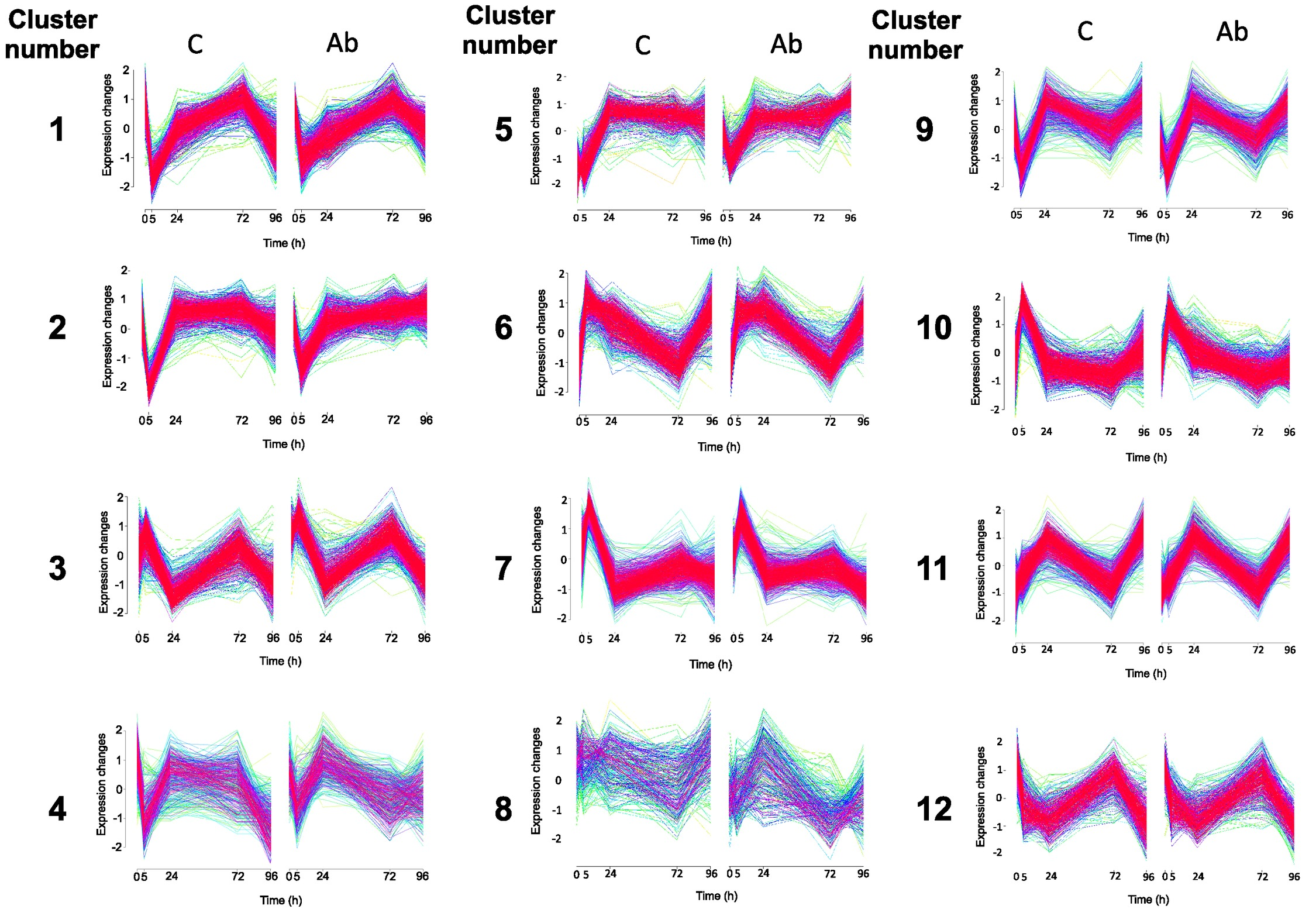

Supplement: S1 Fig — Clusters were generated in the Mfuzz package. Each line represents one gene in the cluster, with line colour indicating strength of membership in the cluster (red being the strongest membership and green the weakest). C = control, Ab = antibiotics. (TIF) [file ppat.1006391.s006.tif]

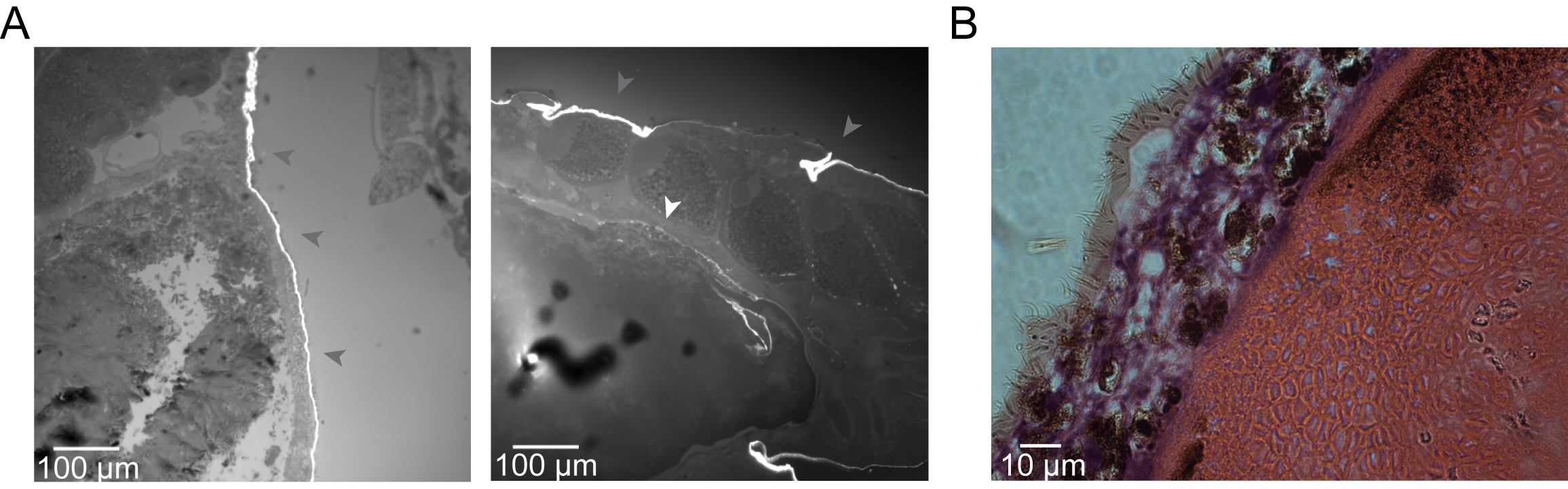

Supplement: S2 Fig — H&E (A) and calcofluor white (B) stained thin sections of engorged midguts 24 h post blood feeding with the addition of 100μM polyoxin D to the blood meal. In (B), arrowheads indicate staining of the cuticle (grey arrows) and the fragmentary peritrophic matrix (white arrows). (TIF) [file ppat.1006391.s007.tif]

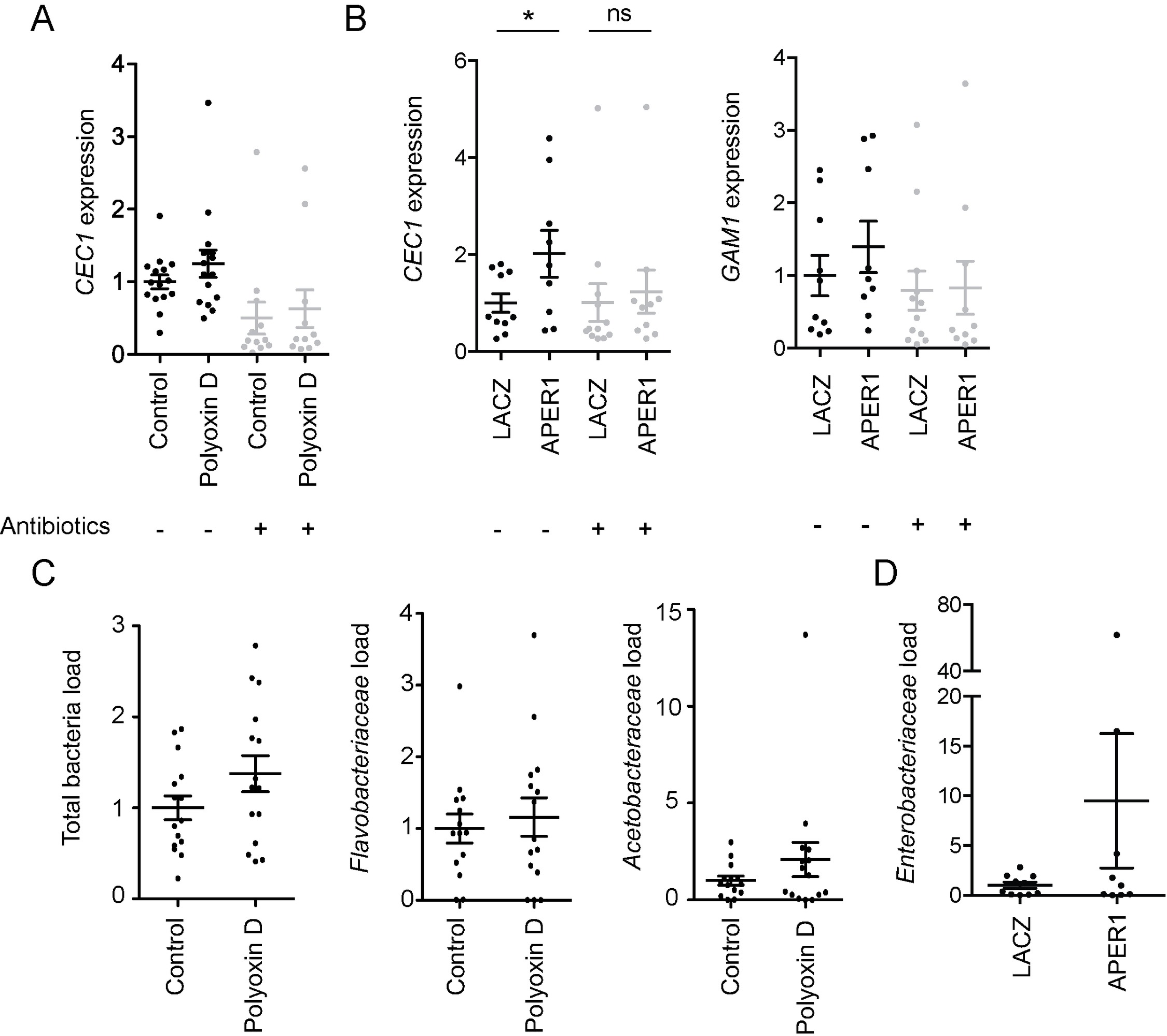

Supplement: S3 Fig — (A) CEC1 expression, relative to AgS7, in the midgut 24h after feeding with a blood meal supplemented with 100μM polyoxin D or an equal volume of water (control), plus or minus antibiotic treatment, as determined by qRT-PCR. (B) CEC1 and GAM1 expression in the midgut of APER1 and LACZ (control) knock down mosquitoes 24h after blood feeding, plus or minus antibiotic treatment, as determined by qRT-PCR. (C) Total bacteria load, Flavobacteriaceae load, and Acetobacteraceae load 24h after feeding with 100μM polyoxin D or a control blood meal, relative to AgS7, as determined by qRT-PCR with family specific or universal 16S primers. (D) Enterobacteraceae load in APER1 or LACZ (control) knock down mosquitoes 24h after blood feeding. A-D: Each dot represents a pool of 8–10 (polyoxin D experiments) or 3–5 (APER1 experiments) guts, derived from 4–5 independent experiments. Ratios are normalized within biological replicates to the mean of the control pools. Mean plus/minus standard error is indicated. Statistical significance was assessed by an ANOVA on a linear mixed effect regression model. ‘*’ p<0.05. (TIF) [file ppat.1006391.s008.tif]

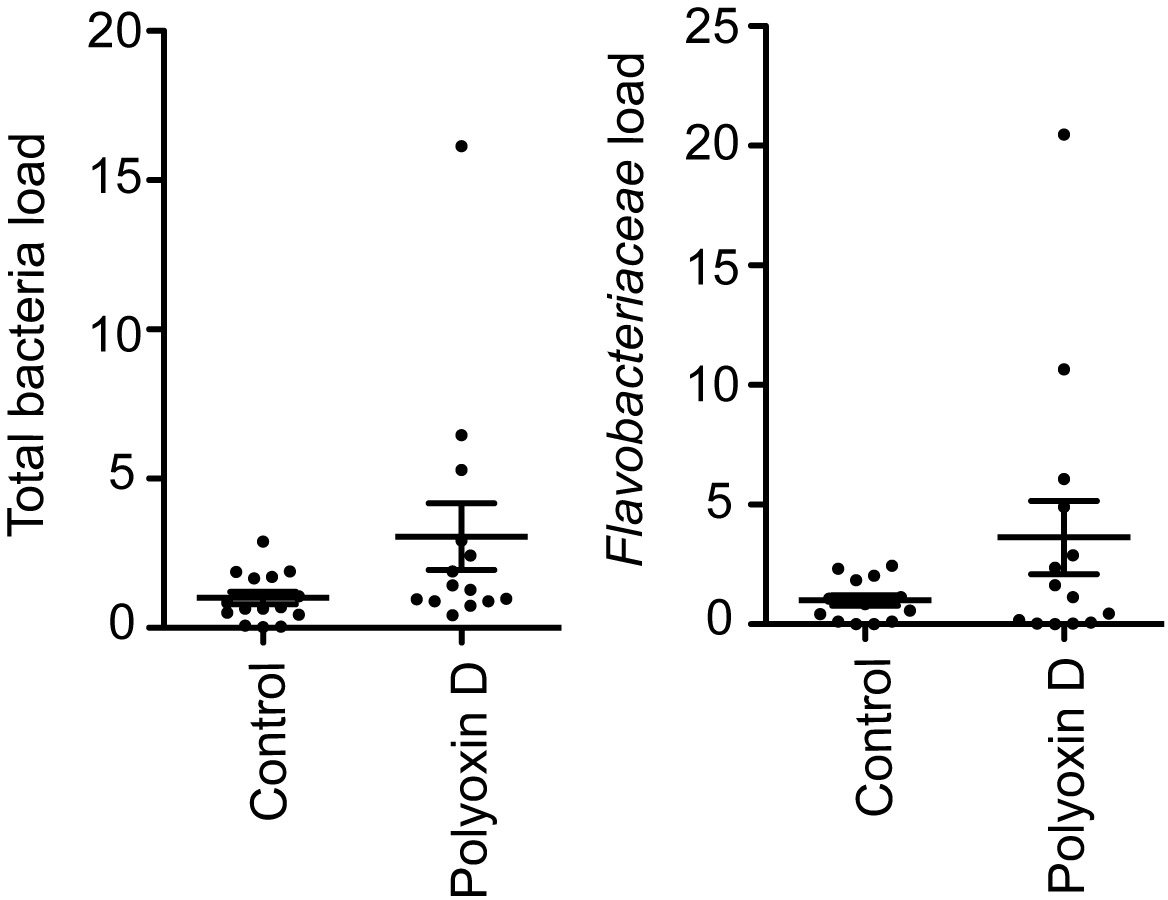

Supplement: S4 Fig — Total bacteria load and Flavobacteriaceae load in the midgut 72 h after feeding with a blood meal supplemented with 100μM polyoxin D or an equal volume of water (control), as determined by qRT-PCR with universal of family-specific 16S primers. Each dot represents a pool of 8–10 guts, derived from 4 independent experiments. Ratios are normalized within biological replicates to the mean of the control pools. Mean plus/minus standard error is indicated. (TIF) [file ppat.1006391.s009.tif]

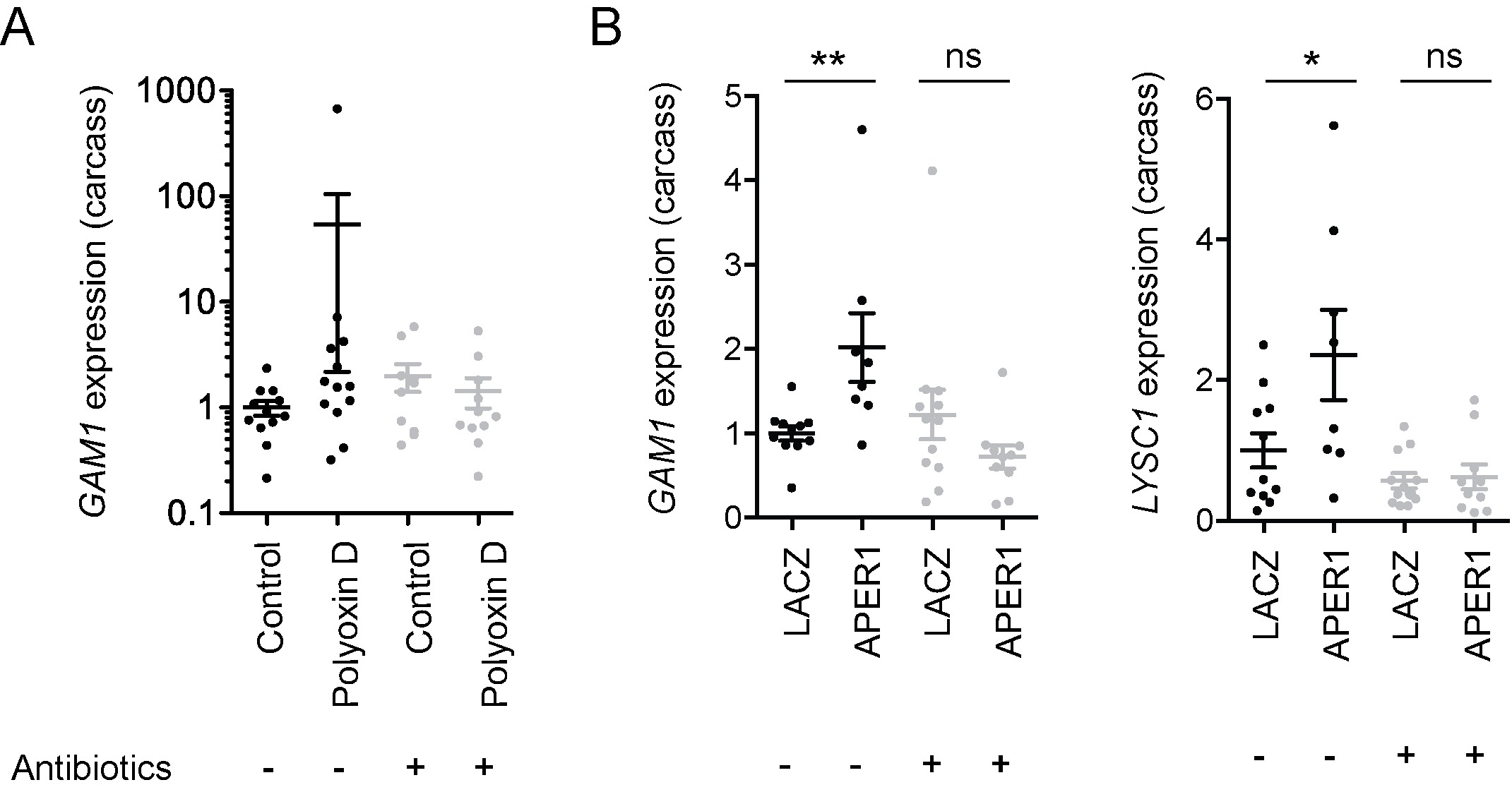

Supplement: S5 Fig — (A) GAM1 expression in the carcass 72 h after feeding with a blood meal supplemented with 100μM polyoxin D or water as a control, plus or minus antibiotic treatment, as determined by qRT-PCR. (B) GAM1 and LYSC1 expression in the carcass of APER1 and LACZ (control) knock down mosquitoes, 24 h after a human blood meal. A-B: Each dot represents a pool of 8–10 (A) or 3–5 (B) carcasses, derived from 4 independent experiments. Ratios are normalized within biological replicates to the mean of the control pools. Mean plus/minus standard error is indicated. (TIF) [file ppat.1006391.s010.tif]

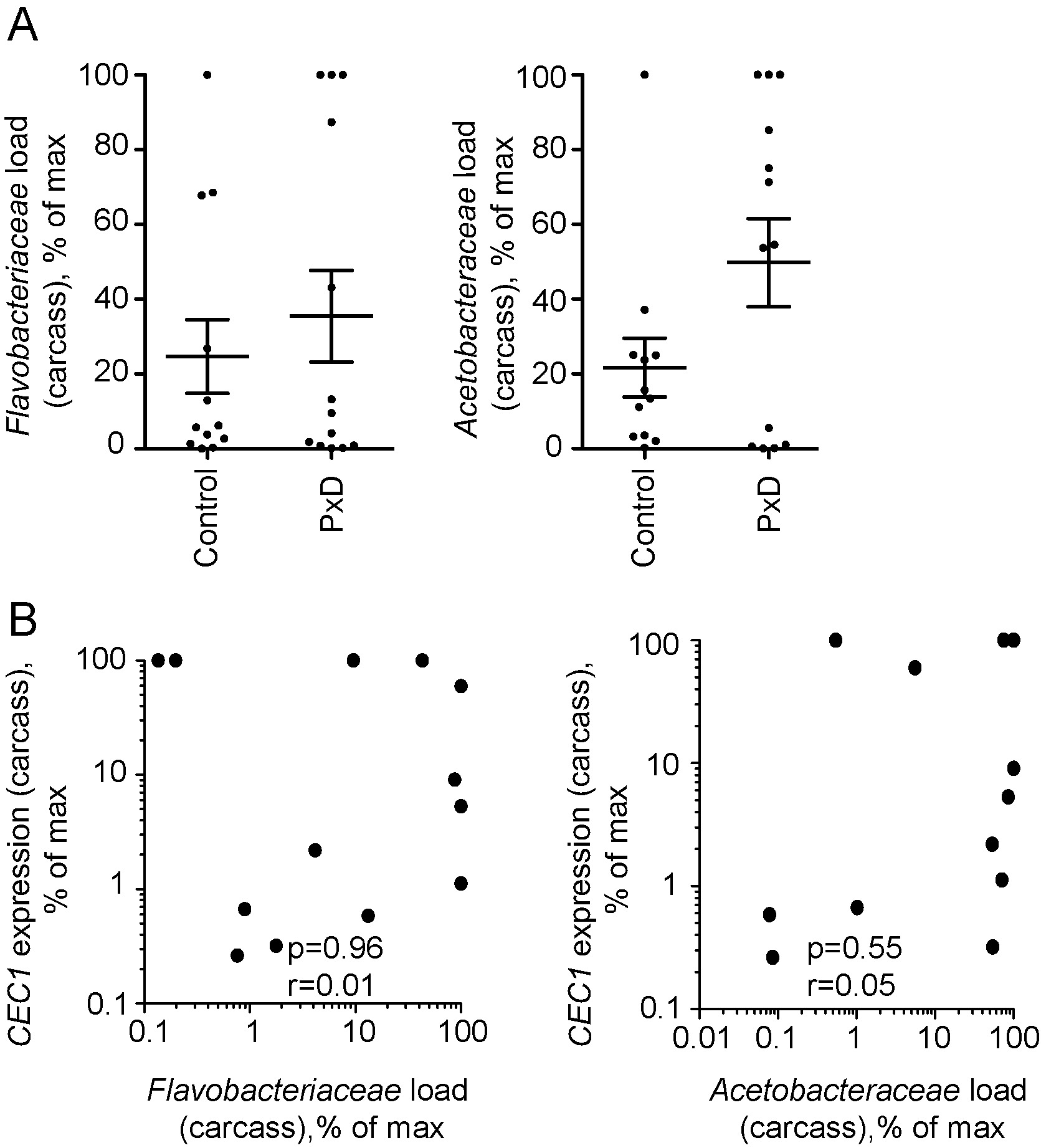

Supplement: S6 Fig — (A) Flavobacteriaceae and Acetobacteraceae load in the carcass 72h after feeding with a blood meal supplemented with 100μM polyoxin D or water as a control. (B) Scatter plots of relative Flavobacteriaceae and Acetobacteraceae load against CEC1 expression in the carcass of polyoxin D fed mosquitoes at 72h post blood feeding. A-B: Ratios are normalized within each biological replicate to the highest value across all conditions (‘100%’). Each dot represents a pool of 8–10 (polyoxin D carcasses, derived from 4 independent experiments. In A, the mean plus/minus standard error is indicated. In B, Spearman’s rank correlation coefficient and associated p-values are indicated. (TIF) [file ppat.1006391.s011.tif]
